# The *Pseudomonas aeruginosa* Tse4 toxin assembles ion-selective and voltage-sensitive ion channels to couple membrane depolarisation with K^+^ efflux

**DOI:** 10.1371/journal.ppat.1012981

**Published:** 2025-06-04

**Authors:** Jessica Rojas-Palomino, Carmen Velázquez, Jon Altuna-Alvarez, Amaia González-Magaña, Maialen Zabala-Zearreta, Matthias Müller, María Queralt-Martín, Antonio Alcaraz, David Albesa-Jové

**Affiliations:** 1 Laboratory of Molecular Biophysics, Department of Physics, University Jaume I, Castellón, Spain; 2 Instituto Biofisika (CSIC, UPV/EHU), Fundación Biofísica Bizkaia/Biofisika Bizkaia Fundazioa (FBB), Leioa, Spain; 3 Departamento de Bioquímica y Biología Molecular, University of the Basque Country, Leioa, Spain; 4 Ikerbasque, Basque Foundation for Science, Bilbao, Spain; Centre National de la Recherche Scientifique, Aix-Marseille Université, FRANCE

## Abstract

*Pseudomonas aeruginosa* employs the Type VI secretion system (T6SS) to outcompete other bacteria in its environment. Among the effectors secreted by the T6SS of *P. aeruginosa* PAO1, Tse4 is known for its potent antibacterial activity. This study elucidates the molecular function of Tse4, which promotes cell depolarization in competing bacteria. Our results show that Tse4 spontaneously incorporates into lipid monolayers and forms multi-ionic channels in planar bilayers, with either ohmic conduction or diode-like rectifying currents and a preference for cations over anions. These observations allow us to propose a model of action whereby Tse4 channels couple cell depolarization with K^+^ efflux. These insights into Tse4’s pore-forming activity enhance our understanding of bacterial competition and exemplify a finely tuned antibacterial strategy, coupling its ability to cause membrane depolarization with potassium efflux that synergises with other T6SS effectors. These results highlight the sophistication of *Pseudomonas aeruginosa*’s competitive arsenal.

## Author summary

*Pseudomonas aeruginosa* is an opportunistic bacterial pathogen known for its sophisticated strategies to dominate its environment and outcompete rival bacteria. One key strategy involves injecting toxic proteins into competing bacteria using the Type VI secretion system (T6SS). In this study, we investigate the molecular details of Tse4, a potent antibacterial toxin delivered by this system. We discovered that Tse4 spontaneously integrates into bacterial membranes, creating tiny, ion-selective channels that allow positively charged ions to flow in and out of the targeted cells. This ion leakage leads to membrane depolarization followed by K^+^ efflux, disrupting essential cellular processes and ultimately leading to bacterial death. Our work provides the first direct biophysical evidence that Tse4 acts as a pore-forming toxin, assembling channels with unique electrical properties and multi-ionic selectivity. Understanding how Tse4 induces ion imbalance and depolarization helps explain its effectiveness in bacterial competition and highlights its remarkable complexity.

## Introduction

Bacterial competition is a fundamental aspect of microbial ecosystems, playing a pivotal role in shaping the dynamics of bacterial communities and influencing ecological and pathogenic processes. Understanding the mechanisms underlying this bacterial competition is of paramount importance, as it unveils the intricate strategies employed by microorganisms to gain a competitive advantage in their habitats. In this context, the present research focuses on elucidating the molecular function of Tse4, a Type VI secretion system exported effector produced by *Pseudomonas aeruginosa*, a versatile opportunistic pathogen known for its ability to adapt, survive, and persist in diverse environments [1].

The Type VI secretion system (T6SS) is a complex molecular machine, structurally and evolutionary related to the bacteriophage tail and spike, that many gram-negative bacteria use to inject effector proteins directly into neighbouring bacterial or eukaryotic cells [2–8]. The *P. aeruginosa* genome contains three independent T6SS clusters (H1, H2, and H3-T6SS) [9,10]. *P. aeruginosa* PAO1 deploys the H1-T6SS in response to an interspecies [11] or intraspecies [12] attack, a crucial role in bacterial competition.

The effectiveness of the *P. aeruginosa* H1-T6SS in bacterial competition lies in the multitude and variety of the antiprokaryotic toxins it injects into competing bacteria. Therefore, unravelling the molecular functions of these toxins is crucial for gaining insights into this bacterial competition mechanism. The H1-T6SS of *P. aeruginosa* PAO1 is known to deliver eight antiprokaryotic toxins, targeting various cellular components and functions. These include (i) the cell wall peptidoglycan, with Tse1 exhibiting peptidase activity [13,14], and Tse3 displaying muramidase activity [15,16]; (ii) NAD(P)+, impacted by Tse6 possessing glycosidase activity [17]; (iii) the DNA, influenced by Tse7 with DNase activity [18]; (iv) protein biosynthesis, modulated by Tse8 [19,20]; and (v) the membrane, affected by Tse4 [6,21] and Tse5 [22,23].

In the present study, we investigate the molecular function of Tse4 (PA2774), one of the most potent antiprokaryotic H1-T6SS effectors of *P. aeruginosa* PAO1 known to date [24]. Tse4 associates with haemolysin co-regulated protein 1 (Hcp1) for H1-T6SS-dependent delivery into target cells [6,25]. Along with its cognate immunity protein Tsi4, Tse4 can also be found in the inner membrane of *P. aeruginosa* [26]. Previous in vivo studies showed that Tse4 induces relatively sophisticated membrane permeabilisation regarding ion specificity and size exclusion [21]. Thus, it was shown that intoxication by Tse4 promoted cell growth inhibition that critically depended on electrolyte composition, being stronger in sodium and lithium chloride than in equimolar solutions of potassium chloride or sucrose. Also, several small solutes between 300 – 700 Da (0.45 – 0.65 nm equivalent hydrodynamic radius) could not access the cytoplasm of Tse4-intoxicated cells. Taking all these results into account, it was hypothesised that Tse4 promotes the formation of relatively narrow ion-selective membrane pores [21]. However, this hypothesis was not verified at the molecular level, probably due to the difficulty in expressing and purifying Tse4 for in-vitro studies [25]. Here, we successfully produced Tse4 by exploiting a novel purification system based on the encapsulation properties of Tse5 [23], demonstrating its potential biotechnological application for expressing toxic and/or hydrophobic proteins and allowing us to perform the first-ever biophysical study of the Tse4 function at the molecular level.

Our data directly demonstrate that Tse4 is a pore-forming toxin that integrates spontaneously into lipid monolayers, forming ohmic or rectifying channels in planar bilayers. Detailed electrophysiological analyses also reveal that Tse4-induced pores exhibit a general preference for cations that can range from mild to substantial selectivity, including a certain degree of chemical specificity. The fact that Tse4-poration activity does not involve detergent-like mechanisms disintegrating the membrane integrity aligns with in vivo data showing that Tse4-induced toxicity causes cell depolarization and bacteriostasis [21]. Remarkably, the finding of non-ohmic Tse4-induced pores that open preferentially at positive potentials suggests that they may operate as outward rectifying channels, potentially playing a role in the modulation of action potentials, as observed with other toxins known to influence membrane excitability [27–29], or contributing to electrical communication in bacterial communities [30].

## Results

### Heterologous expression of a Tse5^ΔCT^-Tse4 chimera

Tse4’s hydrophobicity and toxicity prevented us from producing it in milligram quantities for biophysical studies. To overcome these challenges, we explored the encapsulating capacity of Tse5, a *P. aeruginosa* H1-T6SS-dependent Rearrangement hot spot (Rhs) toxin [6,31]. Over a decade of research into bacterial Rhs toxins has revealed tremendous insight into their structures and mechanisms of action. Examples of bacterial Rhs toxins involved in the intercellular competition include insecticidal toxin complexes (Tc) [32,33], Gram-negative T6SS-associated Rhs toxins [34–36], and the Gram-positive wall-associated protein A (WapA) [37].

Rhs polymorphic toxins contain tyrosine/aspartate repeats (YD-repeats) [38] that assemble into a β-sheet that spirals to form a barrel-like/cocoon structure [34, 35, 39–41]. A recent cryo-EM structure revealed that Tse5 is organised in three fragments [23]. The N-terminal fragment contains a proline-alanine-alanine-proline (PAAR)−like motif that presumably targets the effector to the T6SS. This fragment results from auto-cleavage between Lys47 and Pro48 residues by a yet-to-be-discovered mechanism. The central fragment assembles this cocoon (Tse5-Shell), encapsulating the C-terminal toxic fragment (Tse5-CT). Cleavage of the toxic fragment is vital for toxin activation. This cleavage is mediated by a conserved aspartyl protease domain characterised by a DPxGx_19_DPxG motif and located at the C-terminal end of the Tse5-Shell fragment [23]. The toxicity of Tse5-CT is attributed to the assembly in the cytoplasmic membrane of competing bacteria of ion-selective proteolipidic pores. This ion channel activity of Tse5-CT causes cell depolarization and, ultimately, bacterial death [22].

Based on Tse5’s capacity to encapsulate the pore-forming toxin Tse5-CT, we decided to investigate whether Tse5-Shell could be used to encapsulate Tse4, which contains several predicted transmembrane regions (S1 Fig). The Tse5-Shell’s cavity volume is ~ 32,000 Å^3^, which should be sufficient to encapsulate the 19.2 kDa Tse4 protein, which has a calculated dry volume of 23,491 Å^3^.

**Fig 1:**
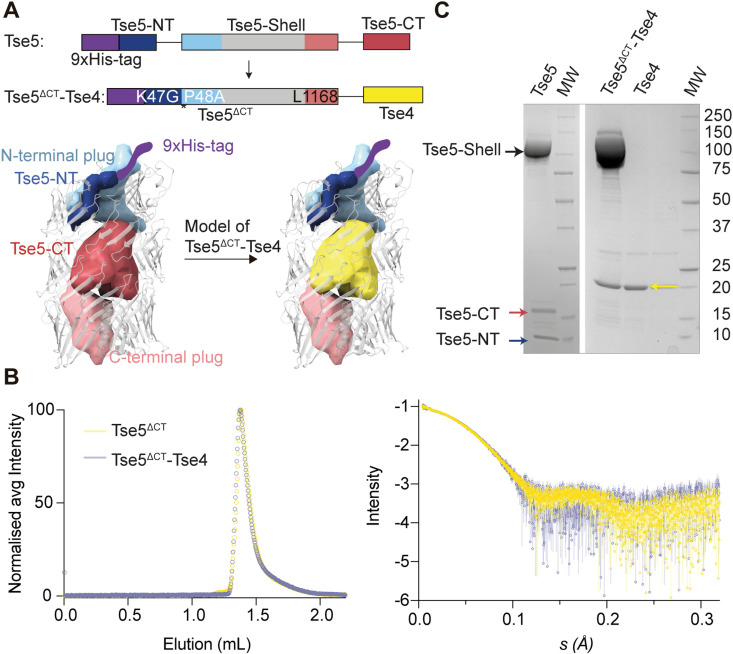
Heterologous expression of a Tse5^ΔCT^-Tse4 chimera. A. The top panel shows a schematic representation of Tse5 and Tse5^ΔCT^-Tse4 constructs. Tse4 replaces the Tse5-CT fragment. The Tse5^ΔCT^-Tse4 construct contains a double point mutation (K47G-P48A) that inhibits cleavage between residues G47 and A48. The bottom panel shows a predicted 3D structure of Tse5 and Tse5^ΔCT^-Tse4 constructs. The construct is designed so that Tse4 is encapsulated inside the Tse5 shell/cocoon structure. B. SEC-SAXS analysis of Tse5 and Tse5^ΔCT^-Tse4 proteins. The left panel shows the normalised SEC signal profile for both proteins, indicating they have very similar elution properties. The right panel plot represents the SAXS data for the two proteins. C. SDS-PAGE of purified wild-type Tse5 (left) showing that WT Tse5 auto-cleaves, resulting in three fragments, Tse5-Shell, Tse5-CT and Tse5-NT. On the right is the SDS-PAGE of Tse5^ΔCT^-Tse4 and Tse4 following its separation from the Tse5^ΔCT^ fragment.

To this end, we have engineered a chimeric protein based on Tse5, where the Tse4 sequence replaces its Tse5-CT fragment (Figs 1A and S2). Tse4 is inserted after Leu1168 residue of Tse5, which is where the aspartyl protease domain of the Tse5-Shell cleaves. The chimeric Tse5^ΔCT^-Tse4 protein contains a poly-His tag at the N-terminus for protein purification by Ni-NTA affinity chromatography, and it also contains mutations K47G and P48A that inhibit auto-cleavage of its N-terminal fragment. Remarkably, size exclusion chromatography-small angle X-ray scattering (SEC-SAXS) indicates Tse5-CT deletion mutant (Tse5^ΔCT^) and Tse5^ΔCT^-Tse4 have comparable hydrodynamic behaviour, as indicated by having comparable SEC elution profiles and radius of gyration (*R*_g _= 36.5 Å and 36.2 Å for Tse5^ΔCT^ and Tse5^ΔCT^-Tse4, respectively, as calculated from the Pair distance distribution function [*P*(*r*)]; Table 1 and Fig 1B). Furthermore, when running Tse5^ΔCT^-Tse4 on a denaturing sodium dodecyl sulphate-polyacrylamide gel electrophoresis (SDS-PAGE), Tse4 appears as a fragment around the ~ 20 kDa protein marker, indicating the Tse5^ΔCT^ aspartyl protease domain remains active (Fig 1C). Following Tse5^ΔCT^-Tse4 expression and purification, we can separate Tse4 from Tse5^ΔCT^ by denaturation with 6 M urea and precipitation with ammonium phosphate (Fig 1C, see Methods section for experimental details). Altogether, this data indicates that Tse4 is cleaved and remains encapsulated inside Tse5^ΔCT^.

**Table 1:** Summary of SEC-SAXS data analysis for Tse5^ΔCT^ and Tse5^ΔCT^-Tse4.

Data-collection parameters	Tse5^ΔCT^	Tse5^ΔCT^-Tse4
Instrument	Beamline B21 (DLS)	Beamline B21 (DLS)
Wavelength (Å)	0.946	0.946
Strategy	SEC-SAXS	SEC-SAXS
*q* range (Å^-1^) ^*b*^	0.0134-0.2179	0.0143-0.2215
Exposure time (sec/frame)	3	3
Injected concentration (mg·mL^-1^)	1.8	1.2
Temperature (K)	288	288
Structural parameters*		
*I*(0) (cm^-1^) [from Guinier]	0.088 ± 7.5e^-05^	0.052 ± 6.4e^-05^
*R*_g_ (Å) [from Guinier]	36.71 ± 0.05	36.11 ± 0.07
*I*(0) (cm^-1^) [from P(*r*)]	0.088 ± 5.5e^-05^	0.052 ± 6.4e^-05^
*R*_g_ (Å) [from P(*r*)]	36.46 ± 0.03	36.23 ± 0.06
*d*_max_ (Å)	109.24	117.31
Porod volume estimate (Å^3^)	204,875	145,775
Dry volume calculated from sequence (Å^3^)	156,753	180,243
**Molecular-mass determination by Bayesian inference**		
MW Estimate (Da)	138,225	138,225
MW Probability (%)	48.13	50.88
Credibility (Da)	127,450 – 151,450	127,450 – 151,450
Credibility Interval Probability (%)	98.25	99.34
Calculated monomeric from sequence (Da)	133,223	152,322
**Software employed**		
Primary data reduction	GDA	GDA
Data processing	CHROMIXS/PRIMUS	CHROMIXS/PRIMUS

### Tse4 inserts spontaneously into model membranes to assemble narrow membrane pores

Previous cellular studies demonstrated that Tse4 leads to impaired growth in *P. aeruginosa* [21], displaying certain sensitivity to specific monovalent cations, particularly Na^+^ and Li^+^ ions. Also, these previous in vivo results provided evidence that Tse4 disrupts the proton motive force by affecting the membrane potential but not pH homeostasis, allowing the passage of ions while excluding larger molecules [21].

**Fig 2:**
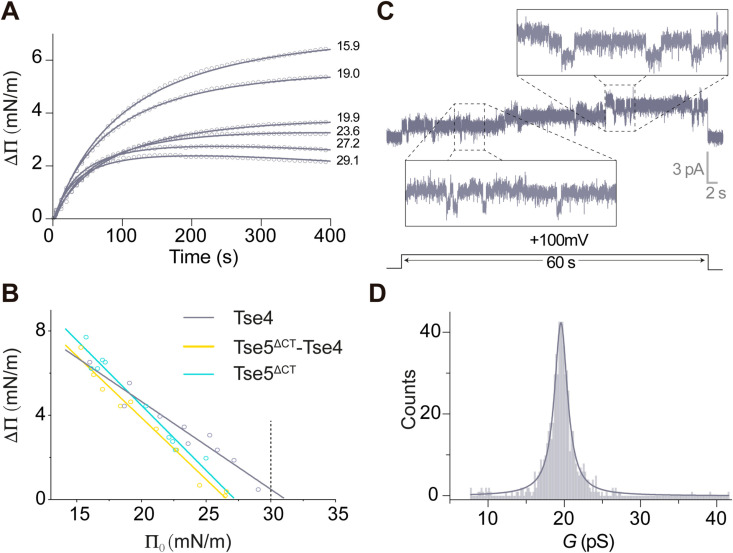
Tse4 inserts spontaneously into model membranes to assemble narrow membrane pores. A. Representative Langmuir-Blodgett balance data showing the lateral pressure increase on lipid monolayers assembled from *E. coli* polar lipid extract (Avanti Polar lipids) after the addition of Tse4 at time 0. Initial lateral pressures (*Π*_0_) in mN m^−1^ for representative experiments are indicated next to each curve. B. Plot of lateral pressure increases (Δ*Π*) as a function of initial lateral pressure (*Π*_0_) for Tse4, Tse5^ΔCT^-Tse4, and Tse5^ΔCT^ (*n* = 10). A maximal insertion pressure (*Π*_c_) of 31.08 (Tse4), 26.53 (Tse5^ΔCT^-Tse4), and 27.17 mN m^−1^ (Tse5^ΔCT^) has been determined by extrapolating the fitted curve to Δ*Π* = 0. The dotted line indicates the threshold value of lateral pressure consistent with unstressed biological membranes. The equations obtained from the linear regression analysis are y = −0.4148*x* + 12.89 (*R*^*2*^ = 0.94), *y* = −0.5832*x* + 15.47 (*R*^2^ = 0.98),*y* = −0.6141*x* + 16.68 (*R*^*2*^ = 0.96) for Tse4, Tse5^ΔCT^-Tse4, and Tse5^ΔCT^, respectively. C. Representative current traces showing small current jumps recorded at a constant voltage of 100 mV in symmetrical 150 mM KCl. D. Frequency distribution analysis of 644 independent current jumps obtained at 100 mV was conducted using a bin width of 0.2 p*S*. The resulting dataset, comprising 170 data points, was fitted to a Gaussian function, indicating a peak in the distribution at 19.6 ± 2.7 pS.

Although the above findings suggested that Tse4 functions by facilitating the formation of ion-selective membrane pores, there was no direct evidence to exclude alternative potential pathways involving other cellular components. To address these questions and provide insight into its molecular mechanism, we first evaluated the capacity of Tse4 to spontaneously partition into the hydrophobic core of lipid monolayers assembled from an *E. coli* polar lipid extract using the Langmuir-Blodgett balance [42] (refer to the Methods section for experimental details). This method detects the insertion of a protein into the hydrophobic core of a monolayer by monitoring the change in lateral pressure (Δ*Π*) from an initial lateral pressure (*Π*_0_). As the initial lateral pressure increases, the rate of protein insertion decreases until it reaches a critical lateral pressure (*Π*_c_) where protein is not able to insert and therefore Δ*Π* = 0. In the biological membrane’s outer monolayer, the lipid packing typically generates lateral surface pressures between 30–35 mN/m [43,44]. Therefore, when the critical lateral pressure falls within this range, it indicates that the protein is spontaneously integrating into the hydrophobic core of the lipid monolayer. The incorporation of Tse4 into lipid monolayers assembled from an *E. coli* polar lipid extract (Avanti Polar lipids) with diverse *Π*_0_ resulted in a critical lateral pressure of 31.1 mN/m, therefore indicating Tse4 spontaneously integrates into the hydrophobic core of the lipid monolayer (Fig 2A and 2B). Remarkably, Tse5^ΔCT^-Tse4 and Tse5^ΔCT^ display similar *Π*_c_ that are below the 30 mN/m threshold, indicating that neither of them spontaneously integrates into the hydrophobic core of the lipid monolayer (*Π*_c_ values for Tse5^ΔCT^-Tse4 and Tse5^ΔCT^ are 26.51 and 27.17 mN/m, respectively).

The capacity of Tse4 to integrate into a model membrane prompted us to investigate its ability to form membrane pores. To this end, we implement a modified solvent-free Montal-Mueller technique [45] (see Methods section for experimental details). First, we assemble a lipid bilayer using the *E. coli* polar lipid extract and a concentration of 150 mM KCl at pH 7.4 on both chambers. Following the addition of 0.4 µM of Tse4 in the *cis* chamber, we observe ion-channel-like activity in the form of relatively stable currents but also exhibiting transitions between different conductive levels with a diversity of lifetimes (Fig 2C). Since from current recordings we cannot discriminate between the collective action of clusters of small units and potential individual wide pores, we considered traces where the current was variable and calculated the conductance increments (ΔG = ΔI/V) associated with each current jump ΔI. As expected from the random nature of current jumps, we obtain a Gaussian distribution with a peak located at G ~ 20 ± 3 pS (Fig 2D), which is comparable to values measured under similar conditions (salt concentration and membrane composition) for channels of known structure such as Gramicidin A (gA) (G ~ 30 pS, r ~ 0.4 nm) [46,47] and the lowest level of the antibiotic peptide Alamethicin, namely L0 (G ~ 50 pS, r ~ 0.7 nm) [48,49]. Hence, an estimation of r ~ 0.5 nm for Tse4-induced pores seems reasonable and agrees satisfactorily with previous studies showing that molecules larger than 300 Da, about 0.5 nm of hydrodynamic radius, were unable to access the cytoplasm of Tse4-intoxicated cells [21].

### Tse4 forms a variety of cation-selective protein pores with mild cation specificity

**Fig 3:**
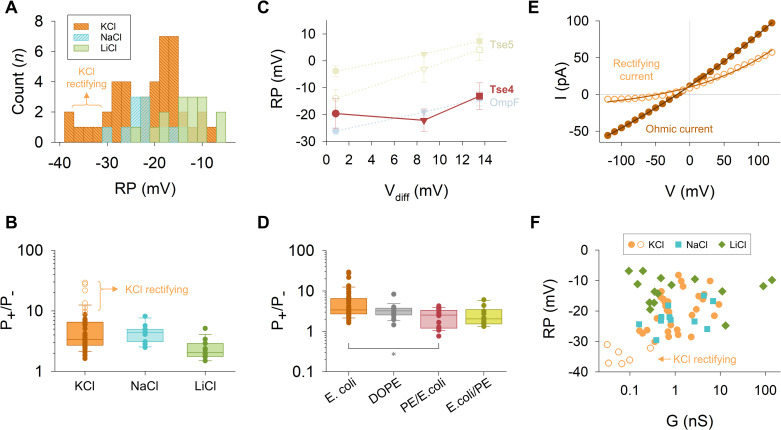
Tse4 forms a variety of cation-selective pores with mild cation specificity and diverse voltage sensitivity. A. Histograms illustrating the reversal potentials (RP) for Tse4-induced channels using *E. coli* lipid mixture in KCl (orange, n = 43), NaCl (blue, n = 11) or LiCl (green, n = 15). The frequency distribution analysis was performed with a bin width of 2 mV. Rectifying pores in KCl display the most negative RP. B. Box and whiskers plot displaying the P_+_/P_-_ ratios in KCl, NaCl and LiCl derived from the experiments presented in panel A. Here and elsewhere, the boundary of the box closest to zero indicates the 25^th^ percentile, a line within the box marks the median, and the boundary of the box farthest from zero indicates the 75^th^ percentile. Whiskers (error bars) above and below the box indicate the 90^th^ and 10^th^ percentiles. Rectifying pores in KCl exhibit the maximum P_+_/P_-_ values. C. RP measured for Tse4 in *E. coli* lipids and compared with Tse5 and OmpF in KCl (circles), NaCl (triangles) or LiCl (squares) plotted versus the corresponding diffusion potential (V_diff_) for each salt. For Tse5, results are shown for charged (solid symbols) and neutral (open symbols) lipids. RP for Tse4 in KCl only includes ohmic channels, as there are no rectifying currents in other salts. For Tse4, data are means of n = 37 (KCl), 11 (NaCl) and 15 (LiCl); for Tse5 in charged lipid n = 15 (KCl), 13 (NaCl) and 10 (LiCl); for Tse5 in neutral lipid n = 34 (KCl), 18 (NaCl) and 13 (LiCl); and for OmpF n = 8 (KCl), 6 (NaCl) and 7 (LiCl). n indicates independent channels. D. Box and whiskers plot displaying the P_+_/P_-_ ratios measured in Tse4 in KCl using different lipid compositions, as indicated. Significance was tested using one-way analysis of variance (ANOVA) followed by a Holm-Sidak test for pair-wise comparison. The differences between groups were all non-significant (p > 0.05) except for the difference between *E. coli* and PE/*E. coli* (p = 0.03), indicated with an asterisk. E. Example current-voltage curves depicting the electrical behaviour of ohmic (filled circles) and rectifying (open circles) pores induced by Tse4 in KCl and *E. coli* lipid mixture. The solid lines represent a fit with an empirical equation used to categorize I-V curves as ohmic or rectifying (see Methods for details). F. Scatter plot of the RP as a function of the conductance measured in KCl (filled circles correspond to ohmic currents while open circles indicate rectifying currents), NaCl, or LiCl, derived from the experiments presented in panels A and B. In all panels, experiments were performed in a 250/50 mM salt gradient.

Next, we measured the applied voltage needed to cancel the current (the so-called Reversal Potential or RP) in experiments with Tse4 under a concentration gradient of 250/50 mM of different salts. In all studied salts (KCl, NaCl, LiCl), we see a variety of cation-selective channels (RP < 0 always), as shown in the histograms of Fig 3A. The higher dispersion is found in KCl, where the maximum probability is around RP = −20 mV. Interestingly, the peaks for NaCl and LiCl appear at RP = −22 mV and −13 mV, respectively. Considering that the theoretical limit (Nernst potential setting the ideal maximum selectivity) for 250/50 mM gradient is RP ~ −41mV, our data suggest Tse4-induced membrane pores have a multi-ionic character but with a marked preference for cations. Indeed, this image appears when RP measurements are turned into ion permeability ratios (P_+_/P_-_) using the Goldman-Hodgkin-Katz flux equation (GHK). Tse4-induced pores in model *E. coli* cytoplasmic membranes showed a P_Na_+/P_Cl_^-^ = 4.4 ± 1.6, P_K_+/P_Cl_^-^ = 4.0 ± 1.9 and P_Li_+/P_Cl_^-^ = 2.4 ± 0.9 (Fig 3B). Notice that P_K_+/P_Cl_^-^ = 4.0 ± 1.9 correspond to ohmic pores. As shown in the next section, Tse4 in KCl also induces the formation of non-ohmic channels with a higher selectivity (P_K_+/P_Cl_^- ^~ 19) (indicated as ‘KCl rectifying’ in Fig 3A and 3B).

Concerning a possible channel specificity among cations, note that the ionic selectivity is a property of the system that depends on the channel and the electrolyte solution flowing through it. Consequently, the measured RP includes the diffusion potential (V_diff_) arising from the intrinsic differences in ionic diffusivities between cations and anions [50]. For a 250/50 mM gradient, V_diff_ can be calculated via Planck’s equation using tabulated free solution values of ionic diffusion coefficients [50] yielding V_diff_ ~ 0.8 mV for KCl (note that K^+^ and Cl^-^ ions have very similar diffusion coefficients), V_diff_ ~ 8.6 mV for NaCl, and 13.5 mV for LiCl (note that for the considered gradient all V_diff_ values are positive whereas all measured RP values are negative). If the channel selectivity were non-specific, RP measured for salts of different cations should differ approximately in the difference between their respective V_diff_, as it is the case in the channels formed by Tse5 [51] or from the bacterial porin OmpF of *Escherichia coli* [52]. These channels are considered non-specific and yield an approximate straight line when plotting their RP vs. V_diff_ (Fig 3C). However, this is not the case in Tse4-induced channels: the difference in RP in KCl vs NaCl should be +8 mV, and it is –2 mV. Likewise, the difference in RP in KCl vs LiCl should be +13 mV, but it is only +7 mV, so the plot RP vs. V_diff_ does not show a straight line (Fig 3C). Considering this, we conclude that Tse4-induced pores have a greater channel discrimination for Na^+^ and Li^+^ than for K^+^.

Tse4 channel selectivity may be mainly controlled by the protein characteristics or could otherwise be influenced by the membrane lipid charge, as is the case of a variety of pore-forming proteins such as viroporins [53], cell-penetrating peptides [54–56] or toxins including Tse5 [22,51,57,58]. To test this possibility, we measured the RP of Tse4-induced channels in a neutral lipid, DOPE, and in asymmetric membranes formed of *E. coli* mixture on one side and DOPE on the other side of the membrane (*E. coli*/PE and PE/*E. coli*) (Fig 3D). Although we find a statistically significant difference between *E. coli* and PE/*E. coli*, we believe that it has not relevant biophysical implications, given that we are obtaining cation selective channels similar to that in *E. coli* even in the fully neutral lipid (P_K_+/P_Cl_- = 3.3 ± 1.5) (Fig 3D). Therefore, based on the evidence provided, we presume that Tse4-induced pores are basically protein channels where lipid molecules are not decisively involved in the channel architecture.

### Non-ohmic behaviour and rectification in Tse4 channels and its implications for selectivity and conductance

In selectivity experiments performed with a 250/50 mM salt gradient, we obtain the RP (corresponding to the voltage applied that yields I = 0), and we also compute the full current-voltage (I-V) relationship, where we observe mainly linear I-V curves. However, for KCl in *E. coli* lipid mixtures we detect some that seemed to deviate from linearity, so we formally classify the recorded currents into ohmic (resistor-like conduction) and non-ohmic (asymmetric diode-like conduction) based on an ideality factor (see Methods section for details) (Figs 3E and S3). Our data shows that, out of 43 independent channels, 86% are ohmic (n = 37) and 14% are rectifying (n = 6) (S3 Fig). We hypothesize that the ohmic behavior does not arise from pores inserting in random orientation and thus averaging the voltage sensitivity. This is because all non-linear curves showed rectification in the same direction. Also, some ohmic I-V curves (I4-I6 in S3 Fig) had a lower conductance than some rectifying curves (I38 in S3 Fig). Interestingly, currents that show rectification also display the lowest conductances. Moreover, all non-ohmic pores act as outward rectifying channels, given that they open at positive potentials allowing mostly cation efflux [59]. Rectifying I-V curves are usually associated with structural inhomogeneities in the system, either in terms of pore geometry (i.e., conical pores) or charge distribution [60].

In all cases, the measured RP can be associated with the experimental G obtained as the I/V slope if the curve is ohmic or as the local I/V quotient in the I = 0 region for rectifying I-V curves. For all the electrolytes under investigation, we observe a considerable dispersion in the RP vs G representation (Fig 3F). For KCl and NaCl, one may speculate on a relationship between the greater selectivity and the associated value of G, which would suggest that channel enhanced selectivity (higher values of RP) is attained by narrowing the permeation pathway for ions (lower values of G). Note that if the effect of conductance is only due to the number of functional pores being present in the membrane, the RP remains constant as this quantity does not depend on the number of channels inserted. However, for LiCl we observe a different scenario, where smaller values of G correspond to lower values of RP, indicating that the proximity of protein charges to the pore eyelet is not a relevant factor ruling ion selectivity, but other mechanisms exist, such as some specificity derived from residue-ion interactions [50,61].

The case of KCl is particularly interesting because the high dispersion found in RP values can be rationalized in terms of ohmic and rectifying channels. Thus, while low and moderate RP values correspond to weakly selective (P_K_+/P_Cl_^-^ ≤ 10) ohmic channels (orange filled circles in Fig 3F and 3B), slightly higher RP (P_K_+/P_Cl_^-^ ~ 10–30) values are linked to rectifying channels (open orange circles in Fig 3F and 3B).

## Discussion

### A conceptual model for Tse4-induced changes in cell membrane potential

Potassium is the most abundant intracellular cation in all living organisms, where it is required for numerous basic cellular functions, including regulating intracellular pH, governing the magnitude of the transmembrane electrical potential, and balancing turgor/osmotic pressure [62]. Therefore, the ability of bacteria to regulate intracellular potassium (K⁺) levels is critical for maintaining homeostasis, surviving environmental stresses, and adapting to hostile host environments. Bacteria employ multiple potassium transport systems, each tailored to specific environmental conditions, to achieve this regulation. These systems not only play fundamental physiological roles but also contribute to pathogenesis and antimicrobial resistance [63].

**Fig 4:**
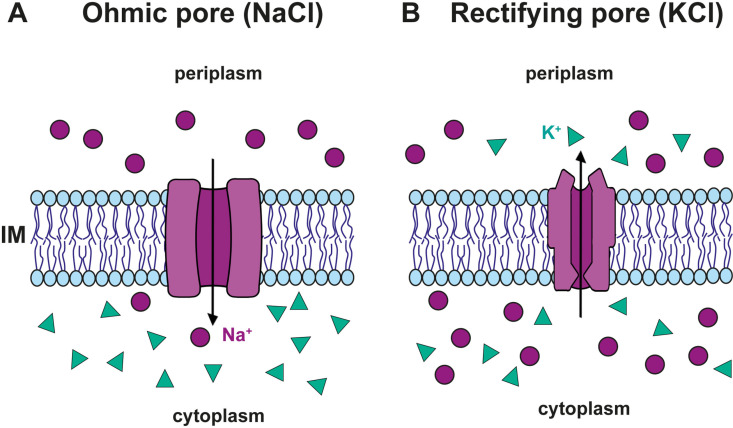
A conceptual model for Tse4-induced changes in cell membrane potential. Tse4 forms ion-selective pores in bacterial membranes, exhibiting mild specificity for Na⁺ and Li⁺ over K ⁺ . These pores facilitate Na⁺ influx (A) and K⁺ efflux (B), driven by concentration gradients and membrane potential. Tse4-induced K⁺ efflux is enhanced under depolarised conditions due to rectifying pore conduction, maintaining cytoplasmic electroneutrality without affecting pH. This model integrates observations of K⁺ efflux, membrane depolarisation, and unchanged pH, highlighting Tse4’s role in disrupting membrane potential and sensitising bacteria to other T6SS effectors.

Previous cellular studies showed that Tse4 induces a potassium flux from the cytoplasm to the extracellular milieu [21]. In principle, such externalisation of positive charge should lead to a hyperpolarised state in which the charge imbalance would dramatically alter the intracellular pH. However, the actual situation was quite different. Membrane depolarization was proven using potential-sensitive fluorescent dyes, and pH-sensitive fluorescent proteins showed no changes in pH in Tse4-intoxicated cells [21]. Given that, we postulate that a more subtle mechanism could operate in which Tse4-induced pores disturb cellular homeostasis by playing a dual role in terms of cation specificity and voltage-dependent conduction as shown in Fig 4.

Our electrophysiological studies demonstrate that Tse4 forms ion-selective pores in bacterial membranes, displaying a relative preference for monovalent cations over anions, including mild Na⁺ and Li⁺ specificity compared to K⁺. These findings correlate well with the previous in vivo work [21], which showed that Tse4 toxicity in *Pseudomonas aeruginosa* depends on the type of extracellular salt, particularly with increased sensitivity to sodium and lithium ions compared to potassium or sucrose. Considering this, we hypothesise that Tse4-induced cell depolarization could be due to the influx of Na^+^ through the ohmic pores created in the membrane (Fig 4A). To justify this premise, we consider not only the cationic selectivity observed in Tse4 experiments (including a slight preference for Na^+^ over K^+^) but also the asymmetric situation of these ionic species in the respective cellular compartments. At resting potential, Na^+^ ions accumulate in the periplasm while being excluded from the cytoplasm due to the action of Na^+^/K^+^-ATPase. Accordingly, in the case of pore formation, both the concentration gradient and the resting potential push Na^+^ ions towards the cytoplasm (Fig 4A), in contrast to K⁺ cations and Cl^-^ anions that are mostly unaffected because the membrane resting potential is relatively close to their respective equilibrium potentials.

Remarkably, we have also observed that in the case of KCl, Tse4-induced pores may exhibit non-ohmic (rectifying) conduction. These rectifying pores open at positive membrane potentials − in vivo this would mean that rectifying Tse4 pores are silent at resting membrane potentials, and become active after cell depolarization. These rectifying pores are highly selective to cations (see Fig 3B) so that they could facilitate K⁺ efflux while rejecting Cl^-^ influx, with the efflux rate increasing in correlation with less negative membrane potentials—consistent with the K^+^ efflux measured in vivo [21].

With the available biophysical and in vivo data, we speculate that, when a few Tse4 molecules are delivered into the target bacterium, some of them form ohmic channels that allow instant Na^+^ influx while others create outward rectifying channels that are opened when the membrane becomes depolarised (Fig 4B). Within this simplified conceptual model, the influx of Na^+^ is balanced in terms of charge by the efflux of K^+^ so that no change in proton concentration or chloride influx is required to maintain electroneutrality in the cytoplasm. As a result, all experimental evidence (K^+^ efflux, membrane depolarisation, and no pH change) could be included in a single unified mode of action.

It should be noted that such a conceptual model may be oversimplified because it focuses only in cation transport disregarding the possibility of direct interactions between Tse4 and other cellular components, such as the endogenous bacterial Na^+^ and K^+^ channels [64,65] or the voltage dependence of the Na^+^/K^+^-ATPase [66]. Furthermore, Tse4 synergises with other effectors to enhance antibacterial activity. Tse4’s disruption of the membrane potential and K^+^ efflux could also explain the observed increased sensitivity of target cells to the activity of other T6SS effectors, such as Tse1 and Tse3, which degrade peptidoglycan, and Tse6, a NAD^+^ glycohydrolase. The loss of the membrane potential and K^+^ efflux could activate proton motive force (PMF)-sensitive autolysins [67], accelerating the structural degradation caused by cell wall-targeting effectors, while concurrently inhibiting PMF-dependent transporters, exacerbating the metabolic stress induced by Tse6 [21].

## Conclusion

In this study, we successfully expressed and purified the Tse5^ΔCT^-Tse4 chimera, overcoming the inherent challenges posed by the hydrophobicity and toxicity of Tse4. This was achieved using the encapsulating properties of Tse5, which allowed us to isolate Tse4 for further biophysical and electrophysiological studies. By employing this chimeric expression system, we have investigated the pore-forming activity of Tse4 in vitro and its role in bacterial competition. Our results show that Tse4 spontaneously incorporates into lipid monolayers and forms multi-ionic channels in planar bilayers, showing either ohmic conduction or diode-like rectifying currents with mild to significant cation selectivity. Based on this, we hypothesise that Tse4-induced pores disturb cellular homeostasis by playing a dual role in terms of cation specificity and voltage-dependent conduction.

## Methods

### Construct design, protein expression and purification

The gene coding for Tse4 (PA2774) was synthesised by GenScript (GenScript, NJ, USA) and cloned into a pET29a(+) vector (pet29a(+)::K47G-P48A) that derives from the parental vector pet29a(+)::9xhis-Tse5. The last plasmid includes, between the NdeI and HindIII restriction sites, a 5’ 9xHis-tag and a tobacco etch virus protease cleavage site (ATGGGCAGCAGCCATCATCATCATCATCATCATCATCACAGCAGCGGCGAAAACCTGTATTTTCAGGGCGGATCC), followed by the coding sequence of Tse5 (PA2684). To avoid N-terminal cleavage of Tse5, a GenScript derived mutation led to the new parental vector pet29a(+)::K47G-P48A, containing two single point mutations of residues K47 and P48 to glycine and alanine, respectively. The protein sequence of Tse4 was cloned into pet29a(+)::K47G-P48A exchanging Tse5-CT toxin sequence. The final pet29a(+)::*tse5*^ΔCT^-*tse4* plasmid codes for the construct Tse5^ΔCT^-Tse4 (S1 Table and S1 Data).

*Escherichia coli* Lemo21(DE3) cells were transformed with pET29a(+)::*tse5*^ΔCT^*-tse4* plasmid and grown overnight at 37 °C in shaking conditions within flasks containing 200 mL of LB media supplemented with 50 μg/mL kanamycin, 34 μg/mL chloramphenicol and 2 mM rhamnose. For Tse5^ΔCT^-Tse4 overexpression, bacterial cultures were diluted to OD_600_ value of 0.1 with 2 L of fresh LB medium supplemented with both antibiotics at the same concentrations, but lacking rhamnose. When cells reached an OD_600_ value of 0.7 after growing at 37 °C in shaking conditions, protein expression was induced by adding isopropyl β-D-1-thiogalactopyranoside (IPTG) at the final concentration of 1 mM. Bacterial cultures were then left overnight with agitation at 18 °C. Finally, cells were pelleted and stored at −80 °C for later use.

Pellet from 2 L of bacterial culture was resuspended in 30 mL of 50 mM Tris–HCl pH 8.0, 500 mM NaCl, 20 mM imidazole (solution A) with 4 μL of benzonase endonuclease (Millipore, Sigma) and a tablet of protease inhibitor cocktail (cOmplete, EDTA-free, Roche). Cells were then disrupted by sonication for 3 minutes (continual cycles of 10 s ON and 59 s OFF with 60% amplitude), and the suspension was ultra-centrifuged for 60 min at 125748 x g. The supernatant was filtered using a 0.2 μm syringe filter and then loaded into a HisTrap HP column of 5 mL (GE Healthcare) equilibrated with solution A to perform an immobilised metal affinity chromatography on a fast protein liquid chromatography system (ÄKTA FPLC; GE Healthcare). The column was washed with solution A at 0.3 mL/min until the value of absorbance at 280 nm was almost zero. Tse5^ΔCT^-Tse4 was eluted with 100% of 50 mM Tris–HCl pH 8, 500 mM NaCl and 500 mM imidazole (solution B) at 2 mL/min. Peak fractions were pooled, and protein was injected into a HiLoad Superdex 200 26/600 pg, previously equilibrated with 20 mM Tris–HCl pH 8, 150 mM NaCl and 2 mM DTT. Tse5^ΔCT^-Tse4 eluted as a single monodispersed protein but SDS–PAGE showed two major protein fragments, Tse5^ΔCT^ and Tse4 (Fig 1B and 1C). The identity of the Tse4 band was confirmed by mass spectrometry (S2 Data). Fractions containing Tse5^ΔCT^-Tse4 (checked by SDS-PAGE) were pooled, and concentration was assessed by measuring absorbance at 280 nm (*ca.* yield: 30 mg/L).

Using Amicon centrifugal filter units of 30 kDa molecular mass cut-off (Millipore), Tse5^ΔCT^-Tse4 was concentrated up to 17 mg mL^−1^. To separate the two fragments, protein denaturation was achieved by diluting the concentrated protein in solution A with 8 M urea to a final concentration of 6 M. Once the protein was incubated for 30’ under constant shaking, it was loaded with a peristaltic pump (Cytiva) into a HisTrap HP column of 5 mL (GE Healthcare), previously washed and equilibrated with solution A containing 6 M urea. The column was washed with 20 mL of solution A at 0.3 mL/min, and unbound fractions (flowthrough) containing Tse4 were collected. Elution was then performed with 10 mL of solution B at 2 mL/min to recover the Tse5^ΔCT^ fragment. Flowthrough containing only Tse4 was precipitated with 1.5 M ammonium sulphate and 15 min centrifugation at 15000 × g. Precipitated Tse4 was washed twice with MiliQ, flash frozen with liquid nitrogen, lyophilised and then stored at −80 °C until use. The purity of the protein was confirmed by SDS–PAGE (Fig 1C).

### Study the insertion of Tse5^ΔCT^-Tse4 and Tse4 in lipid monolayers using the Langmuir–Blodgett balance technique

The value of the critical lateral pressure (*Π*_c_) of Tse5^ΔCT^-Tse4 and Tse4 was measured by the Langmuir–Blodgett balance technique with a DeltaPi-4 Kibron tensiometer (Helsinki, Finland) to check whether an insertion into a lipid monolayer happens spontaneously. The temperature in each experiment was set at 25 °C using a water bath (JULABO F12). *E. coli* polar lipid extract (Avanti Polar lipids) dissolved in chloroform at 1 mg/mL was extended with a Hamilton microsyringe over 1.25 mL of the aqueous surface (5 mM Hepes pH 7.4, 150 mM NaCl) previously added into each circular trough (Kibron μTrough S system, Helsinki, Finland) of 2 cm in diameter. Starting from different initial surface pressure (*Π*_0_) of the lipid monolayer, within the interval of 15–30 mN/m, changes in surface pressure (ΔΠ) were recorded when the protein was injected into the aqueous subphase at a final concentration of 0.4 μM. Tse5^ΔCT^-Tse4 was dissolved in 20 mM Tris-HCl pH 8.0, 150 mM NaCl, 2 mM DTT, injecting the buffer alone as a control. Tse4 was dissolved in dimethyl sulfoxide (DMSO), so the control was performed by injecting DMSO alone. When the different ΔΠ were plotted as a function of Π_0_, data was fitted into a linear regression model to obtain the *Π*_c_ by extrapolation (y value when x = 0).

### Electrophysical study of the pore-forming activity for Tse4, Tse5 and OmpF in planar lipid bilayers

Planar lipid bilayers were formed by using a solvent-free modified Montal-Mueller [45] technique. In summary, bilayers were formed by apposition of lipid monolayers on a  ~ 150-μm-diameter hole within a 15-μm-thick Teflon film, separating two symmetrical compartments of a Teflon chamber: *cis* and *trans* (1.8 mL of internal volume each). 3% solution of hexadecane in pentane was used for hole pre-treatment. Membranes were formed from a natural polar lipid extract from *E. coli*, dioleoyl-phosphatidylethanolamine (DOPE) or a combination of both for Tse4 experiments, as indicated in each figure legend. In experiments with Tse5 and OmpF shown in Fig 3C, lipids used were pure DOPE (Tse5, neutral lipid), a combination of dioleoyl-phosphoglycerol (DOPG) and DOPE at a ratio DOPE/DOPG 70:30 w/w (Tse5, charged lipid) or diphytanoyl phosphatidylcholine (DPhPC) (OmpF). All lipids were purchased from Avanti Polar Lipids (S3 Data). The chamber compartments were filled with KCl, NaCl or LiCl solutions at various concentrations indicated in each figure legend. All solutions were buffered by 5 mM Hepes at pH 7.4. After membrane formation and before protein addition, the absence of channel-like activity was verified by monitoring for 10 min with holding voltages ranging from -100 to +100 mV without detecting any non-zero current.

For experiments with Tse4, the protein dissolved in DMSO at a stock concentration of 2 mg/mL was added to the aqueous phase at one (*cis*) side of the bilayer to reach a final concentration of 0.4 µM. Tse5 and OmpF experiments shown in Fig 3C were carried out as described previously [51,68].

Electrical connections were made using a pair of silver/silver chloride electrodes (Ag/AgCl) with 1.5% agarose/2 M KCl bridges. Voltage was applied to the *cis* compartment and grounding was applied to the *trans* compartment. Thus, positive voltages indicate that the *cis*-side is positive with respect to *trans*. To minimize the impact of external noise sources, a double metal screen surrounded the bilayer chamber (Amuneal Manufacturing Corp., Philadelphia, PA). Furthermore, an anti-vibration table (Technical Manufacturing Corp. (TMC), Peabody, MA) was employed to shield the system from potential disruptions caused by mechanical vibrations. Ion channel currents were recorded at room temperature in the voltage-clamp mode using an Axopatch 200B amplifier (Molecular Devices, Sunnyvale, CA). The current was filtered at 10 kHz with an in-line low-pass 8-pole Bessel filter. The data was then digitized at a sampling rate of 50 kHz using a Digidata 1440A (Molecular Devices, Sunnyvale, CA) and transferred to a PC for analysis by pClamp 10 software (Molecular Devices, Sunnyvale, CA).

To generate current-voltage (I-V) plots, holding voltages of variable durations ranging from -120 mV to +120 mV in 10 mV steps were applied to the membrane. The amplitudes of current jumps at each applied voltage were quantified by fitting histograms of current values using a single Gaussian function.

To formally classify Tse4-currents measured with KCl in *E. coli* as ohmic or rectifying, we fitted the I-V plots with the following empirical equation used in Materials Science to analyze currents from semiconductor p/n diodes [69]:


$$I\propto \left[\exp\left(-\frac{eV}{nk_{b}T}\right)-1\right],$$

where *e* is the elementary charge, *k*_*B*_ is the Boltzmann constant, T is the absolute temperature and *n* is an ideality factor. The lower the ideality factor (in absolute value, |*n*|), the more rectifying is the I-V curve. For instance, typical non-ohmic I-V curves from ideal semiconductor diodes yield |*n*| ~ 1–2 and similar values are obtained when applied to rectifying protein ion channels (|*n*| ~ 1–6) [68–71]. Thus, we categorized an I-V curve from Tse4 as rectifying when |*n*| < 10. Moreover, the sign of *n* indicates the type of rectification, with n < 0 for outward rectifying (zero or low negative currents for negative potentials, high positive currents for positive potentials) and n > 0 for inward rectifying (high negative currents for negative potentials, zero or low positive currents for positive potentials) channels.

Conductance G was obtained from the slope of each I-V plot, using the entire curve for ohmic currents and the local slope in the I = 0 region for rectifying currents.

Selectivity was evaluated by measuring the reversal potential (RP, voltage at which the current is cancelled) in experiments under a salt concentration gradient. RP was obtained either from the I-V relationship (determined by the horizontal-axis intercept) or by manually cancelling the observed current. Cation-to-anion permeability ratios were calculated from the RP values with the GHK equation [72].

### Statistics and reproducibility

Statistical analyses were performed using GraphPad Prism 9.5 and SigmaPlot (Systat Software, Inc.), and are detailed in the figure legends.

## Supporting information

S1 Table List of bacterial strains and plasmids used in this study and their characteristics, applications, and origins. (PDF)

S1 Fig Transmembrane prediction of Tse4 using TMHMM - 2.0. (TIF)

S2 Fig The Tse5^ΔCT^-Tse4 protein sequence derived for producing Tse4. Poly-His-tag and the tobacco etch virus protease cleavage site are highlighted in grey shading. The sequence of Tse5^ΔCT^ includes the Tse5-NT (dark blue), and the Tse5-Shell. The latter includes the N-terminal plug (light blue), the YD-repeats that assemble the barrel-like structure (black), and the C-terminal plug (salmon). Mutations K47G-P48A that inhibit cleavage of the Tse5-NT are underscored. The Tse4 sequence is highlighted in yellow.
(TIF)

S3 FigTse4-induced ohmic and rectifying I-V curves measured with KCl in *E. coli* lipid mixtures.Each I-V curve (red circles) correspond to an independent Tse4 insertion (n = 43, indicated as I1-I43). Solid lines correspond to the fitting of equation $I\propto \left[\exp\left(-\frac{eV}{nk_{b}T}\right)-1\right]$ (see Methods for details). The ideality factor *n* and R² are shown for each I-V curve. Insertions 35–40 yield |*n*| < 10, thus being classified as non-ohmic, while the rest are ohmic, with |*n*| > 10. Moreover, the rectifying currents all have a negative *n*, indicating that they correspond to outward rectifying channels (see Methods for details).(PDF)

S4 Fig Uncropped and unedited SDS-PAGE gels. (TIF)

S1 Data The Tse5^ΔCT^-Tse4 protein sequence derived for producing Tse4. Poly-His-tag and the tobacco etch virus protease cleavage site are highlighted in bold. The sequence of Tse5^ΔCT^ includes the Tse5-NT (dark blue), and the Tse5-Shell. The latter includes the N-terminal plug (light blue), the YD-repeats that assemble the barrel-like structure (black), and the C-terminal plug (salmon). Mutations K47G-P48A that inhibit cleavage of the Tse5-NT are underscored. The Tse4 sequence is highlighted in green.
(XLSX)

S2 Data Mass spectrometry of Tse4 after final purification. The table summarizes peptide identification results obtained from LC-MS/MS. Tse4 was identified with high confidence, achieving 100% coverage with 19 unique peptides detected. Detailed data includes peptide sequences, post-translational modifications (notably oxidation and carbamidomethylation), the ion score, molecular weight (kDa), calculated isoelectric point (pI), and retention times (RT). Peptide spectrum matches (PSMs) and charge states confirm robust detection and identification of the Tse4 protein.
(XLSX)

S3 Data List of essential materials employed during the experimental procedures. The table provides detailed information, including chemical names, supplier companies, catalog references, and the specific applications of each material. Materials such as IPTG, LB Miller medium, antibiotics (chloramphenicol, kanamycin), and L-Rhamnose were utilized primarily for bacterial growth and protein expression purposes.
(XLSX)

S4 Data Source Data file. The data plotted in Figs 1–3 is provided. The S4 Data file includes the underlying data used to generate the main figure panels: Figs 1B, 2B–2D and 3A–3F. Each tab in the Excel spreadsheet corresponds to a specific panel and contains the raw or processed data used to create the respective plots. Specifically, Fig 1B includes normalized SEC elution profiles and SAXS intensity data for Tse5 and Tse5^ΔCT^-Tse4 proteins. Fig 2B contains lateral pressure increase values as a function of initial pressure for Tse4, Tse5^ΔCT^-Tse4, and Tse5^ΔCT^. Fig 2C includes representative current traces showing discrete current jumps recorded at a constant voltage of 100 mV in symmetrical 150 mM KCl. Fig 2D presents a frequency distribution of current jumps in symmetrical KCl, fitted to a Gaussian function. Fig 3A–3F comprise electrophysiological data on Tse4-induced channels, including reversal potentials, conductance values, ion selectivity ratios (P_⁺_/P_⁻_), and current-voltage relationships across various salt conditions (KCl, NaCl, LiCl) and lipid compositions.
(XLSX)

## References

[1] Moradali MF, Ghods S, Rehm BHA. *Pseudomonas aeruginosa* Lifestyle: a paradigm for adaptation, survival, and persistence. Front Cell Infect Microbiol. 2017;7. 10.3389/fcimb.2017.00039 PMC5310132 28261568

[2] Pukatzki S, Ma AT, Sturtevant D, Krastins B, Sarracino D, Nelson WC, et al. Identification of a conserved bacterial protein secretion system in Vibrio cholerae using the Dictyostelium host model system. Proc Natl Acad Sci U S A. 2006;103(5):1528–33. doi: 10.1073/pnas.0510322103 16432199 PMC1345711

[3] Mougous JD, Cuff ME, Raunser S, Shen A, Zhou M, Gifford CA, et al. A virulence locus of *Pseudomonas aeruginosa* encodes a protein secretion apparatus. Science. 2006;312(5779):1526–30. doi: 10.1126/science.1128393 16763151 PMC2800167

[4] Aschtgen MS, Gavioli M, Dessen A, Lloubès R, Cascales E. The SciZ protein anchors the enteroaggregative Escherichia coli Type VI secretion system to the cell wall. Molecular Microbiology. 2010;75(4):886–99. 20487285 10.1111/j.1365-2958.2009.07028.x

[5] Basler M, Pilhofer M, Henderson GP, Jensen GJ, Mekalanos JJ. Type VI secretion requires a dynamic contractile phage tail-like structure. Nature. 2012;483(7388):182–6. doi: 10.1038/nature10846 22367545 PMC3527127

[6] Whitney JC, Beck CM, Goo YA, Russell AB, Harding BN, De Leon JA, et al. Genetically distinct pathways guide effector export through the type VI secretion system. Mol Microbiol. 2014;92(3):529–42. doi: 10.1111/mmi.12571 24589350 PMC4049467

[7] Lossi NS, Manoli E, Förster A, Dajani R, Pape T, Freemont P, et al. The HsiB1C1 (TssB-TssC) complex of the *Pseudomonas aeruginosa* type VI secretion system forms a bacteriophage tail sheathlike structure. Journal of Biological Chemistry. 2013;288(11):7536–48. 23341461 10.1074/jbc.M112.439273 PMC3597794

[8] Shneider MM, Buth SA, Ho BT, Basler M, Mekalanos JJ, Leiman PG. PAAR-repeat proteins sharpen and diversify the type VI secretion system spike. Nature. 2013;500(7462):350–3. 23925114 10.1038/nature12453 PMC3792578

[9] Filloux A, Hachani A, Bleves S. The bacterial type VI secretion machine: yet another player for protein transport across membranes. Microbiology (N Y). 2008;154(6):1570–83. 10.1099/mic.0.2008/016840-0 18524912

[10] Boyer F, Fichant G, Berthod J, Vandenbrouck Y, Attree I. Dissecting the bacterial type VI secretion system by a genome wide in silico analysis: what can be learned from available microbial genomic resources?. BMC Genomics. 2009;10:104. doi: 10.1186/1471-2164-10-104 19284603 PMC2660368

[11] Basler M, Ho BT, Mekalanos JJ. Tit-for-tat: type VI secretion system counterattack during bacterial cell-cell interactions. Cell. 2013;152(4):884–94. doi: 10.1016/j.cell.2013.01.042 23415234 PMC3616380

[12] George M, Narayanan S, Tejada-Arranz A, Plack A, Basler M. Initiation of H1-T6SS dueling between *Pseudomonas aeruginosa*. mBio. 2024;15(8):e0035524. doi: 10.1128/mbio.00355-24 38990002 PMC11323562

[13] Hood RD, Singh P, Hsu F, Güvener T, Carl MA, Trinidad RRS, et al. A type VI secretion system of *Pseudomonas aeruginosa* targets a toxin to bacteria. Cell Host Microbe. 2010;7(1):25–37. doi: 10.1016/j.chom.2009.12.007 20114026 PMC2831478

[14] Russell AB, Hood RD, Bui NK, LeRoux M, Vollmer W, Mougous JD. Type VI secretion delivers bacteriolytic effectors to target cells. Nature. 2011;475(7356):343–7. doi: 10.1038/nature10244 21776080 PMC3146020

[15] Li L, Zhang W, Liu Q, Gao Y, Gao Y, Wang Y, et al. Structural Insights on the bacteriolytic and self-protection mechanism of muramidase effector Tse3 in *Pseudomonas aeruginosa*. J Biol Chem. 2013;288(42):30607–13. doi: 10.1074/jbc.C113.506097 24025333 PMC3798531

[16] Lu D, Shang G, Zhang H, Yu Q, Cong X, Yuan J, et al. Structural insights into the T6SS effector protein Tse3 and the Tse3-Tsi3 complex from *Pseudomonas aeruginosa* reveal a calcium-dependent membrane-binding mechanism. Mol Microbiol. 2014;92(5):1092–112. doi: 10.1111/mmi.12616 24724564

[17] Whitney JC, Quentin D, Sawai S, LeRoux M, Harding BN, Ledvina HE, et al. An interbacterial NAD(P)(+) glycohydrolase toxin requires elongation factor Tu for delivery to target cells. Cell. 2015;163(3):607–19. doi: 10.1016/j.cell.2015.09.027 26456113 PMC4624332

[18] Pissaridou P, Allsopp LP, Wettstadt S, Howard SA, Mavridou DAI, Filloux A. The *Pseudomonas aeruginosa* T6SS-VgrG1b spike is topped by a PAAR protein eliciting DNA damage to bacterial competitors. Proc Natl Acad Sci U S A. 2018;115(49):12519–24. doi: 10.1073/pnas.1814181115 30455305 PMC6298103

[19] Nolan LM, Cain AK, Clamens T, Furniss RCD, Manoli E, Sainz-Polo MA, et al. Identification of Tse8 as a type VI secretion system toxin from *Pseudomonas aeruginosa* that targets the bacterial transamidosome to inhibit protein synthesis in prey cells. Nature Microbiology. 2021;6(9):1199–210. 10.1038/s41564-021-00950-8 PMC7611593 34413503

[20] González-Magaña A, Sainz-Polo MÁ, Pretre G, Çapuni R, Lucas M, Altuna J, et al. Structural insights into *Pseudomonas aeruginosa* Type six secretion system exported effector 8. J Struct Biol. 2020;212(3):107651. doi: 10.1016/j.jsb.2020.107651 33096229

[21] LaCourse KD, Peterson SB, Kulasekara HD, Radey MC, Kim J, Mougous JD. Conditional toxicity and synergy drive diversity among antibacterial effectors. Nat Microbiol. 2018;3(4):440–6. 29459733 10.1038/s41564-018-0113-y PMC5876133

[22] González-Magaña A, Altuna J, Queralt-Martín M, Largo E, Velázquez C, Montánchez I, et al. The *P. aeruginosa* effector Tse5 forms membrane pores disrupting the membrane potential of intoxicated bacteria. Commun Biol. 2022;5(1):1189. doi: 10.1038/s42003-022-04140-y 36335275 PMC9637101

[23] González-Magaña A, Tascón I, Altuna-Alvarez J, Queralt-Martín M, Colautti J, Velázquez C, et al. Structural and functional insights into the delivery of a bacterial Rhs pore-forming toxin to the membrane. Nat Commun. 2023;14(1):7808. doi: 10.1038/s41467-023-43585-5 38016939 PMC10684867

[24] Rudzite M, Subramoni S, Endres RG, Filloux A. Effectiveness of *Pseudomonas aeruginosa* type VI secretion system relies on toxin potency and type IV pili-dependent interaction. PLoS Pathog. 2023;19(5):e1011428. doi: 10.1371/journal.ppat.1011428 37253075 PMC10281587

[25] Howard SA, Furniss RCD, Bonini D, Amin H, Paracuellos P, Zlotkin D. The breadth and molecular basis of hcp-driven type vi secretion system effector delivery. mBio. 2021;12(3). 10.1128/mBio.00262-21 PMC8262886 34061601

[26] Casabona MG, Vandenbrouck Y, Attree I, Couté Y. Proteomic characterization of *Pseudomonas aeruginosa* PAO1 inner membrane. Proteomics. 2013;13(16):2419–23. doi: 10.1002/pmic.201200565 23744604

[27] Akanda N, Molnar P, Stancescu M, Hickman JJ. Analysis of toxin-induced changes in action potential shape for drug development. J Biomol Screen. 2009;14(10):1228–35. doi: 10.1177/1087057109348378 19801532 PMC3829631

[28] Gilchrist J, Olivera BM, Bosmans F. Animal toxins influence voltage-gated sodium channel function. Handb Exp Pharmacol. 2014;221:203–29. doi: 10.1007/978-3-642-41588-3_10 24737238 PMC5453185

[29] Rojas-Palomino J, Gómez-Restrepo A, Salinas-Restrepo C, Segura C, Giraldo MA, Calderón JC. Electrophysiological evaluation of the effect of peptide toxins on voltage-gated ion channels: a scoping review on theoretical and methodological aspects with focus on the Central and South American experience. J Venom Anim Toxins Incl Trop Dis. 2024;30:e20230048. doi: 10.1590/1678-9199-JVATITD-2023-0048 39263598 PMC11389830

[30] Prindle A, Liu J, Asally M, Ly S, Garcia-Ojalvo J, Süel GM. Ion channels enable electrical communication in bacterial communities. Nature. 2015;527(7576):59–63. doi: 10.1038/nature15709 26503040 PMC4890463

[31] Hachani A, Allsopp LP, Oduko Y, Filloux A. The VgrG proteins are ‘à la carte’ delivery systems for bacterial type VI effectors. Journal of Biological Chemistry. 2014;289(25):17872–84. 24794869 10.1074/jbc.M114.563429 PMC4067218

[32] Sheets J, Aktories K. Insecticidal Toxin Complexes from Photorhabdus luminescens. In: Ffrench-Constant RH, editor. The Molecular Biology of Photorhabdus Bacteria [Internet]. 2016. p. 3–23. 10.1007/82_2016_55 28233068

[33] Gatsogiannis C, Merino F, Prumbaum D, Roderer D, Leidreiter F, Meusch D, et al. Membrane insertion of a Tc toxin in near-atomic detail. Nat Struct Mol Biol. 2016;23(10):884–90. doi: 10.1038/nsmb.3281 27571177

[34] Jurėnas D, Rosa LT, Rey M, Chamot-Rooke J, Fronzes R, Cascales E. Mounting, structure and autocleavage of a type VI secretion-associated Rhs polymorphic toxin. Nat Commun. 2021;12(1):6998. doi: 10.1038/s41467-021-27388-0 34853317 PMC8636562

[35] Günther P, Quentin D, Ahmad S, Sachar K, Gatsogiannis C, Whitney JC, et al. Structure of a bacterial Rhs effector exported by the type VI secretion system. PLoS Pathog. 2022;18(1):e1010182. doi: 10.1371/journal.ppat.1010182 34986192 PMC8765631

[36] Ma J, Sun M, Dong W, Pan Z, Lu C, Yao H. PAAR‐Rhs proteins harbor various C‐terminal toxins to diversify the antibacterial pathways of type VI secretion systems. Environ Microbiol [Internet]. 2017 Jan 18;19(1):345–60. doi: 10.1111/1462-2920.13621 27871130

[37] Foster SJ. Molecular analysis of three major wall-associated proteins of Bacillus subtilis 168: evidence for processing of the product of a gene encoding a 258 kDa precursor two-domain ligand-binding protein. Mol Microbiol. 1993;8(2):299–310. doi: 10.1111/j.1365-2958.1993.tb01574.x 8316082

[38] Wang YD, Zhao S, Hill CW. Rhs elements comprise three subfamilies which diverged prior to acquisition by Escherichia coli. J Bacteriol. 1998;180(16):4102–10. doi: 10.1128/JB.180.16.4102-4110.1998 9696756 PMC107404

[39] Busby JN, Panjikar S, Landsberg MJ, Hurst MRH, Lott JS. The BC component of ABC toxins is an RHS-repeat-containing protein encapsulation device. Nature. 2013;501(7468):547–50. 23913273 10.1038/nature12465

[40] Li J, Shalev-Benami M, Sando R, Jiang X, Kibrom A, Wang J, et al. Structural Basis for Teneurin Function in Circuit-Wiring: A Toxin Motif at the Synapse. Cell. 2018;173(3):735-748.e15. doi: 10.1016/j.cell.2018.03.036 29677516 PMC5912346

[41] Jackson VA, Meijer DH, Carrasquero M, van Bezouwen LS, Lowe ED, Kleanthous C, et al. Structures of Teneurin adhesion receptors reveal an ancient fold for cell-cell interaction. Nat Commun. 2018;9(1):1079. doi: 10.1038/s41467-018-03460-0 29540701 PMC5851990

[42] Maget-Dana R. The monolayer technique: a potent tool for studying the interfacial properties of antimicrobial and membrane-lytic peptides and their interactions with lipid membranes. Biochim Biophys Acta. 1999;1462(1–2):109–40. doi: 10.1016/s0005-2736(99)00203-5 10590305

[43] Demel RA, Geurts van Kessel WS, Zwaal RF, Roelofsen B, van Deenen LL. Relation between various phospholipase actions on human red cell membranes and the interfacial phospholipid pressure in monolayers. Biochim Biophys Acta. 1975;406(1):97–107. doi: 10.1016/0005-2736(75)90045-0 1174576

[44] Calvez P, Bussières S, Eric Demers, Salesse C. Parameters modulating the maximum insertion pressure of proteins and peptides in lipid monolayers. Biochimie. 2009;91(6):718–33. doi: 10.1016/j.biochi.2009.03.018 19345719

[45] Montal M, Mueller P. Formation of bimolecular membranes from lipid monolayers and a study of their electrical properties. Proc Natl Acad Sci U S A. 1972;69(12):3561–6. 10.1073/pnas.69.12.3561 4509315 PMC389821

[46] Smart OS, Goodfellow JM, Wallace BA. The pore dimensions of gramicidin A. Biophys J. 1993;65(6):2455–60. doi: 10.1016/S0006-3495(93)81293-1 7508762 PMC1225986

[47] Queralt-Martín M, López ML, Aguilella-Arzo M, Aguilella VM, Alcaraz A. Scaling Behavior of Ionic Transport in Membrane Nanochannels. Nano Letters. 2018;18(10): 6604–6610. 10.1021/acs.nanolett.8b03235 30178677 PMC6242701

[48] Aguilella VM, Bezrukov SM. Alamethicin channel conductance modified by lipid charge. Eur Biophys J. 2001;30(4):233–41. doi: 10.1007/s002490100145 11548125

[49] Woolley GA. Channel‐Forming Activity of Alamethicin: Effects of Covalent Tethering. Chem Biodivers. 2007;4(6): 1323–1337. 10.1002/cbdv.200790113 17589884

[50] Alcaraz A, Nestorovich EM, López ML, García-Giménez E, Bezrukov SM, Aguilella VM. Diffusion, exclusion, and specific binding in a large channel: a study of OmpF selectivity inversion. Biophys J. 2009;96(1):56–66. doi: 10.1016/j.bpj.2008.09.024 19134471 PMC2710040

[51] Rojas-Palomino J, Altuna-Alvarez J, González-Magaña A, Queralt-Martín M, Albesa-Jové D, Alcaraz A. Electrophysiological dissection of the ion channel activity of the *Pseudomonas aeruginosa* ionophore protein toxin Tse5. Chem Phys Lipids. 2025;267:105472. doi: 10.1016/j.chemphyslip.2025.105472 39778700

[52] López ML, García-Giménez E, Aguilella VM, Alcaraz A. Critical assessment of OmpF channel selectivity: merging information from different experimental protocols. J Phys Condens Matter. 2010;22(45):454106. doi: 10.1088/0953-8984/22/45/454106 21339594

[53] Alcaraz A, Nieva JL. Viroporins: discovery, methods of study, and mechanisms of host-membrane permeabilization. Q Rev Biophys. 2025;58:e1. 10.1017/S0033583524000192 39806799

[54] Gilbert RJC, Dalla Serra M, Froelich CJ, Wallace MI, Anderluh G. Membrane pore formation at protein-lipid interfaces. Trends Biochem Sci. 2014;39(11):510–6. doi: 10.1016/j.tibs.2014.09.002 25440714

[55] Lee M-T, Hung W-C, Chen F-Y, Huang HW. Mechanism and kinetics of pore formation in membranes by water-soluble amphipathic peptides. Proc Natl Acad Sci U S A. 2008;105(13):5087–92. doi: 10.1073/pnas.0710625105 18375755 PMC2278198

[56] Perini DA, Aguilella-Arzo M, Alcaraz A, Perálvarez-Marín A, Queralt-Martín M. Dynorphin A induces membrane permeabilization by formation of proteolipidic pores. Insights from electrophysiology and computational simulations. Comput Struct Biotechnol J. 2021;20:230–40. doi: 10.1016/j.csbj.2021.12.021 35024095 PMC8718563

[57] Gurnev PA, Nestorovich EM. Channel-forming bacterial toxins in biosensing and macromolecule delivery. Toxins (Basel). 2014;6(8):2483–540. doi: 10.3390/toxins6082483 25153255 PMC4147595

[58] Parker MW, Feil SC. Pore-forming protein toxins: from structure to function. Prog Biophys Mol Biol. 2005;88(1):91–142. doi: 10.1016/j.pbiomolbio.2004.01.009 15561302

[59] Cerana R, Colombo R. Inward and Outward Rectifying K+ Channels of the Plasma Membrane have Different Physiological Roles. Journal of Plant Physiology. 1993;142(6):685–8. doi: 10.1016/s0176-1617(11)80902-8

[60] Alcaraz A, Ramírez P, García-Giménez E, López ML, Andrio A, Aguilella VM. A pH-tunable nanofluidic diode: electrochemical rectification in a reconstituted single ion channel. J Phys Chem B. 2006;110(42):21205–9. doi: 10.1021/jp063204w 17048946

[61] Aguilella VM, Queralt-Martín M, Aguilella-Arzo M, Alcaraz A. Insights on the permeability of wide protein channels: measurement and interpretation of ion selectivity. Integr Biol (Camb). 2011;3(3):159–72. doi: 10.1039/c0ib00048e 21132209

[62] Deshpande S, Durdagi S, Noskov SY. Potassium in biological systems. In: Encyclopedia of metalloproteins. New York, NY: Springer New York; 2013. p. 1799–804.

[63] Do EA, Gries CM. Beyond homeostasis: potassium and pathogenesis during bacterial infections. Infect Immun. 2021;89(7). 10.1128/IAI.00766-20 PMC8486168 33875474

[64] Beagle SD, Lockless SW. Unappreciated Roles for K+ Channels in Bacterial Physiology. Trends Microbiol. 2021;29(10):942–50. doi: 10.1016/j.tim.2020.11.005 33288383 PMC9159956

[65] McCusker EC, Bagnéris C, Naylor CE, Cole AR, D’Avanzo N, Nichols CG. Structure of a bacterial voltage-gated sodium channel pore reveals mechanisms of opening and closing. Nat Commun. 2012;3(1):1102. 23033078 10.1038/ncomms2077 PMC3493636

[66] Gadsby DC, Kimura J, Noma A. Voltage dependence of Na/K pump current in isolated heart cells. Nature. 1985;315(6014):63–5. doi: 10.1038/315063a0 2581143

[67] Rice KC, Bayles KW. Death’s toolbox: examining the molecular components of bacterial programmed cell death. Mol Microbiol. 2003;50(3):729–38. doi: 10.1046/j.1365-2958.2003.t01-1-03720.x 14617136

[68] Queralt-Martín M, Perini DA, Alcaraz A. Specific adsorption of trivalent cations in biological nanopores determines conductance dynamics and reverses ionic selectivity. Phys Chem Chem Phys. 2021;23(2):1352–62. doi: 10.1039/d0cp04486e 33367433

[69] Jain A, Kapoor A. A new method to determine the diode ideality factor of real solar cell using Lambert W-function. Solar Energy Materials and Solar Cells. 2005;85(3):391–6. doi: 10.1016/j.solmat.2004.05.022

[70] Gurnev PA, Bezrukov SM. Inversion of membrane surface charge by trivalent cations probed with a cation-selective channel. Langmuir. 2012;28(45):15824–30. doi: 10.1021/la302676t 23088396 PMC4307797

[71] Queralt-Martín M, Peiró-González C, Aguilella-Arzo M, Alcaraz A. Effects of extreme pH on ionic transport through protein nanopores: the role of ion diffusion and charge exclusion. Phys Chem Chem Phys. 2016;18(31):21668–75. doi: 10.1039/c6cp04180a 27464527

[72] Hodgkin L, Katz B, Biological Association M. The effect of sodium ions on the electrical activity of the giant axon of the squid from the laboratory of the. J Physiol. 1949;8:37–77. 10.1113/jphysiol.1949.sp004310 PMC1392331 18128147

