# Supplementary material for: The Pseudomonas aeruginosa Tse4 toxin assembles ion-selective and voltage-sensitive ion channels to couple membrane depolarisation with K+ efflux
Source: PLoS Pathog. 2025 Jun 4;21(6):e1012981. doi: 10.1371/journal.ppat.1012981 (PMC12169545; doi:10.1371/journal.ppat.1012981)
Supplement: S1 Table — (PDF) [file ppat.1012981.s001.pdf]

S1 Table. Strains and Plasmids used in this study.

Strain	Relevant characteristics	Use	Origin
Escherichia coli			
**Lemo21**	BL21(DE3) strain with an extra plasmid harbouring the gene encoding T7 lysozyme, an inhibitor of the T7 RNAP, under control of the well-titratable rhamnose promoter	Heterologous expression of Tse5$^{\Delta CT}$-Tse4 for purification of Tse4	[1]

Plasmids	Relevant characteristics	Use	Origin
**pET29a(+)::*9xhis-Tse5***	Plasmid harbouring a construct based on tse5 and coding for a 9xHis tag and a tobacco etch virus protease cleavage site at the 5' end	Parental vector	[2]
**pET29a(+)::*K47G-P48A***	Plasmid derived from pET29a(+)::*9xhis-Tse5* coding for K47GP48 point mutations	Avoid the proteolysis of the N-terminal domain	[2]
**pET29a(+)::*tse5*$^{\Delta CT}$*-tse4***	Plasmid derived from pET29a(+)::*K47G-P48A* coding the deletion of Tse5-CT and insertion of Tse4 coding sequence	Heterologous expression of Tse5$^{\Delta CT}$-Tse4 in *E. coli* Lemo21 cells for purification of Tse4	This work

References

1. Schlegel S, Löfblom J, Lee C, Hjelm A, Klepsch M, Strous M, Drew D, Slotboom DJ, De Gier JW: **Optimizing membrane protein overexpression in the Escherichia coli strain Lemo21(DE3)**. *J Mol Biol* 2012, **423**:648–659.
2. González-Magaña A, Tascón I, Altuna-Alvarez J, Queralt-Martín M, Colautti J, Velázquez C, Zabala M, Rojas-Palomino J, Cárdenas M, Alcaraz A, et al.: **Structural and functional insights into the delivery of a bacterial Rhs pore-forming toxin to the membrane**. *Nat Commun* 2023, **14**:7808.
