# Supplementary figures and images for: The Pseudomonas aeruginosa Tse4 toxin assembles ion-selective and voltage-sensitive ion channels to couple membrane depolarisation with K+ efflux
Source: PLoS Pathog. 2025 Jun 4;21(6):e1012981. doi: 10.1371/journal.ppat.1012981 (PMC12169545; doi:10.1371/journal.ppat.1012981)

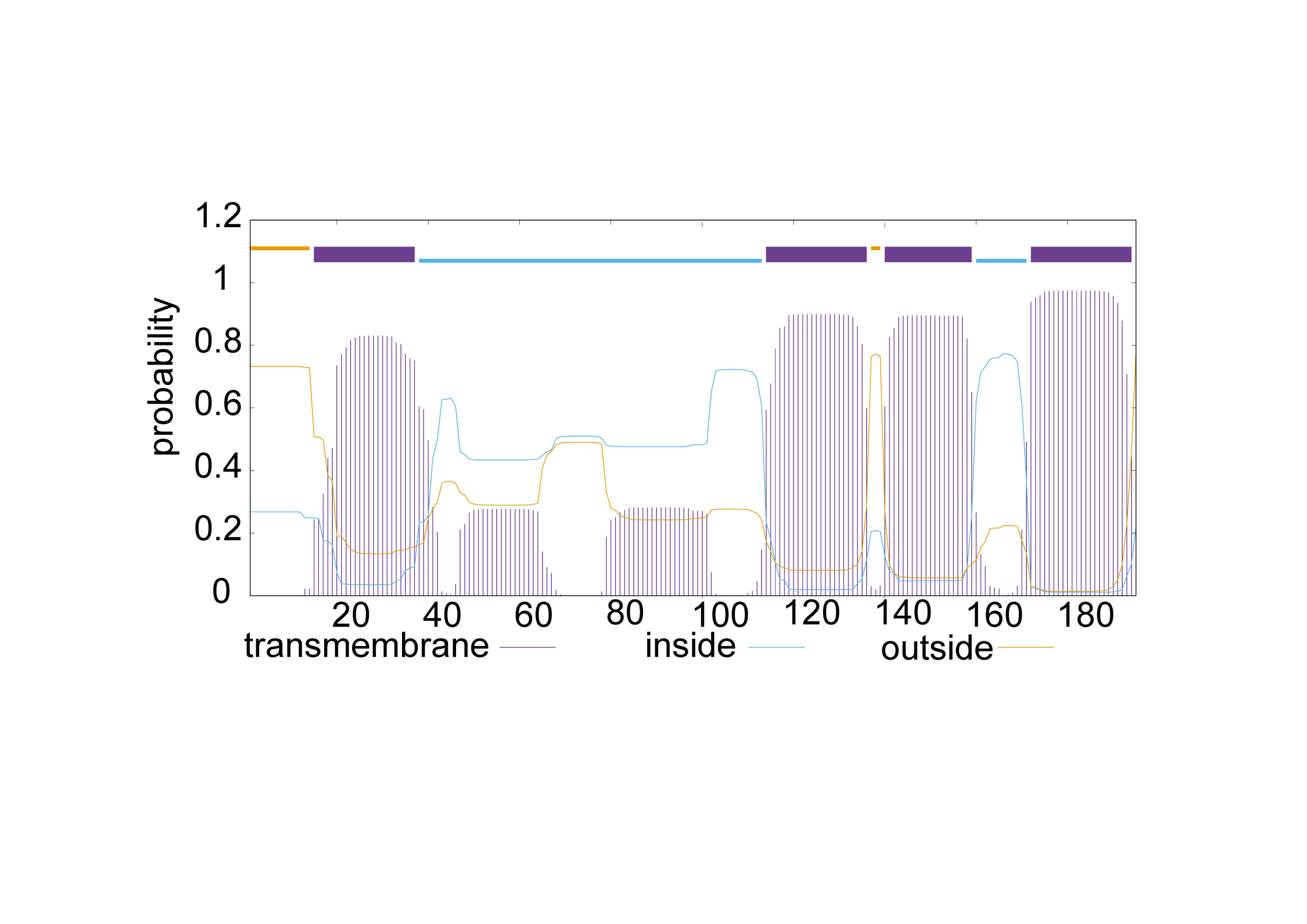

Supplement: S1 Fig — (TIF) [file ppat.1012981.s002.tif]

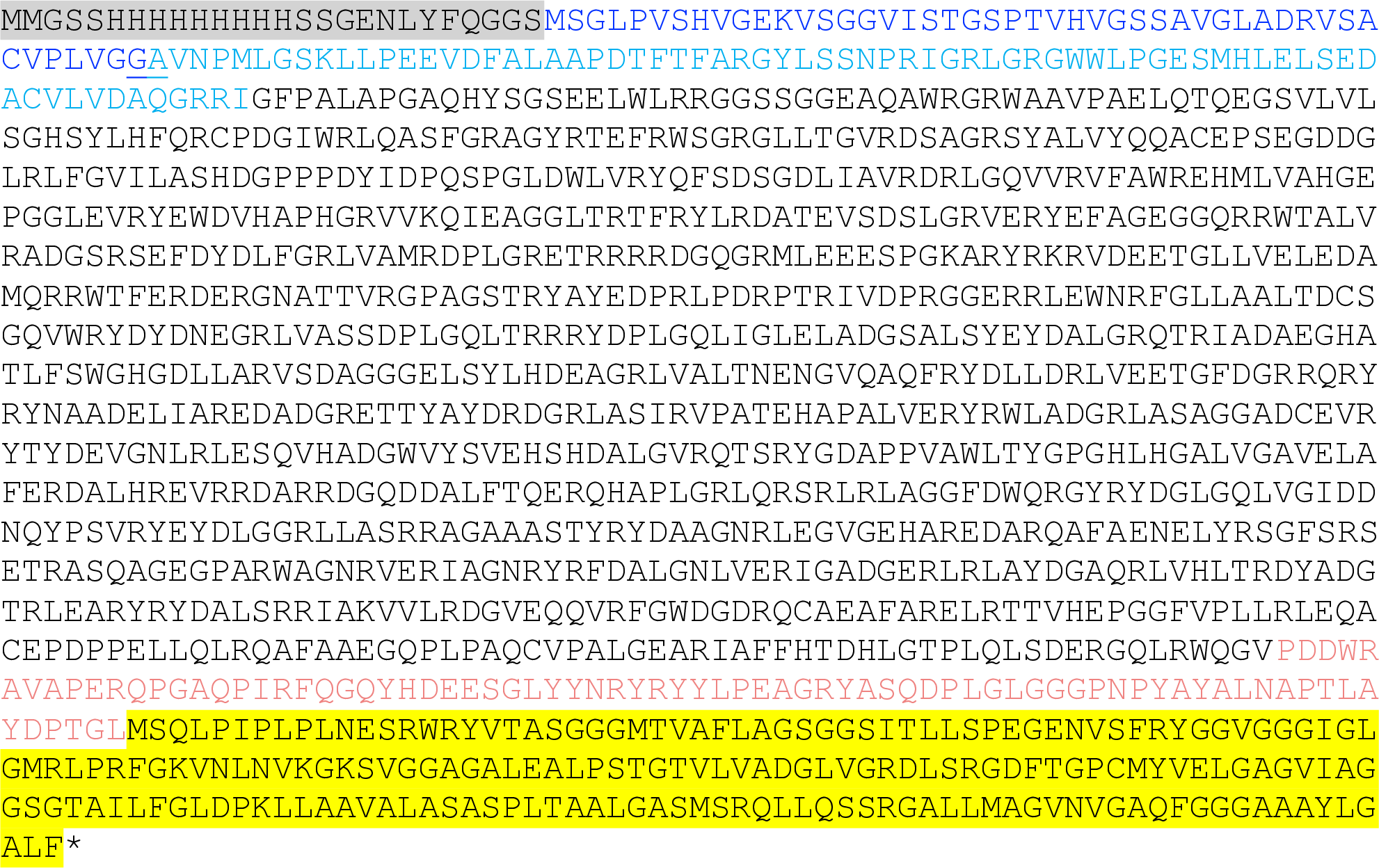

Supplement: S2 Fig — Poly-His-tag and the tobacco etch virus protease cleavage site are highlighted in grey shading. The sequence of Tse5ΔCT includes the Tse5-NT (dark blue), and the Tse5-Shell. The latter includes the N-terminal plug (light blue), the YD-repeats that assemble the barrel-like structure (black), and the C-terminal plug (salmon). Mutations K47G-P48A that inhibit cleavage of the Tse5-NT are underscored. The Tse4 sequence is highlighted in yellow. (TIF) [file ppat.1012981.s003.tif]

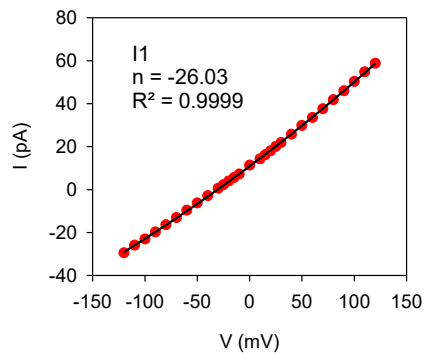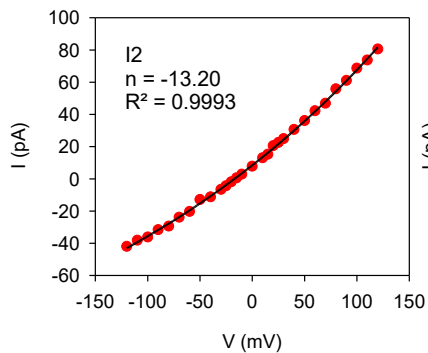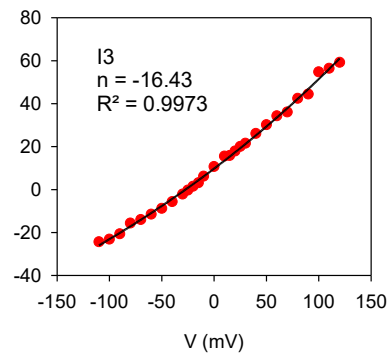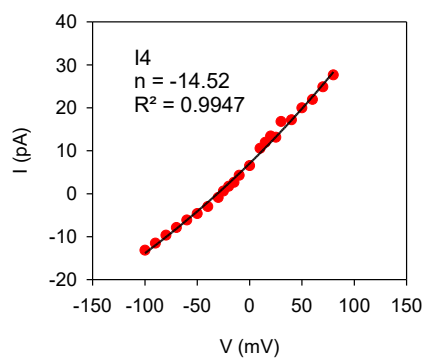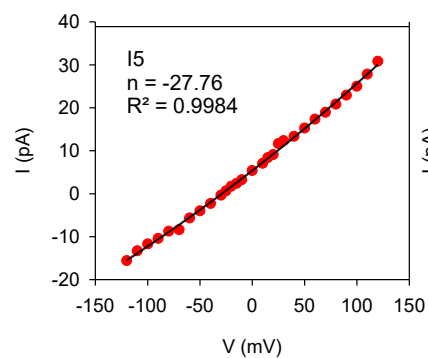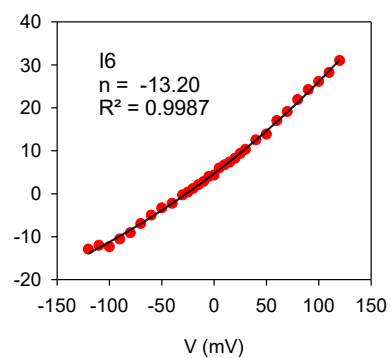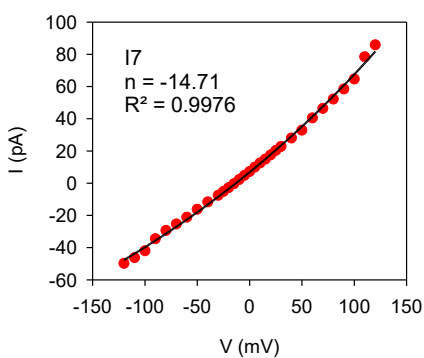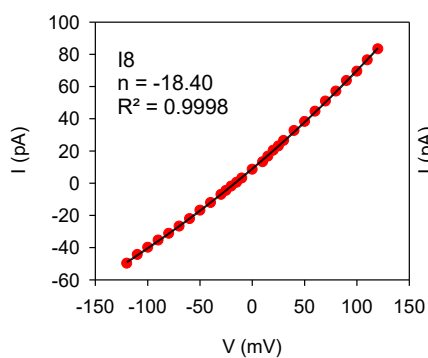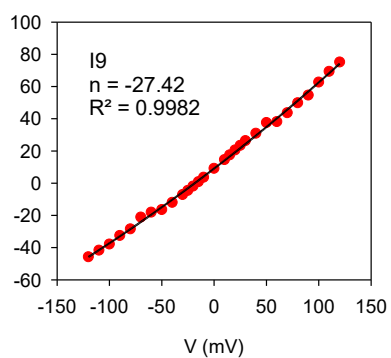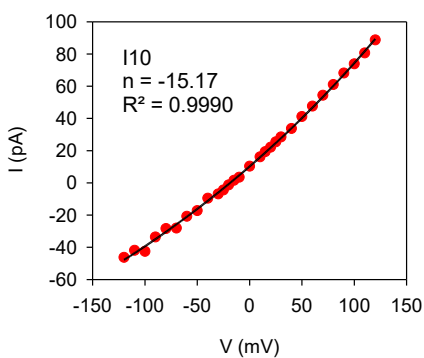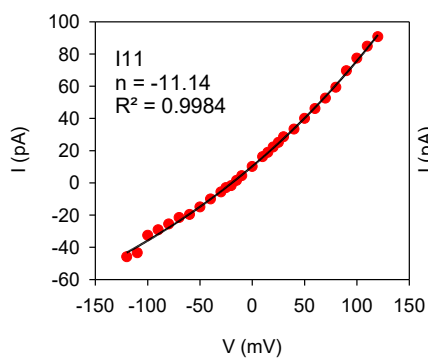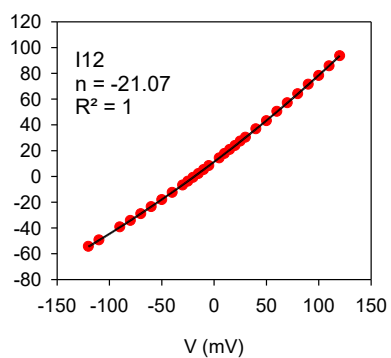

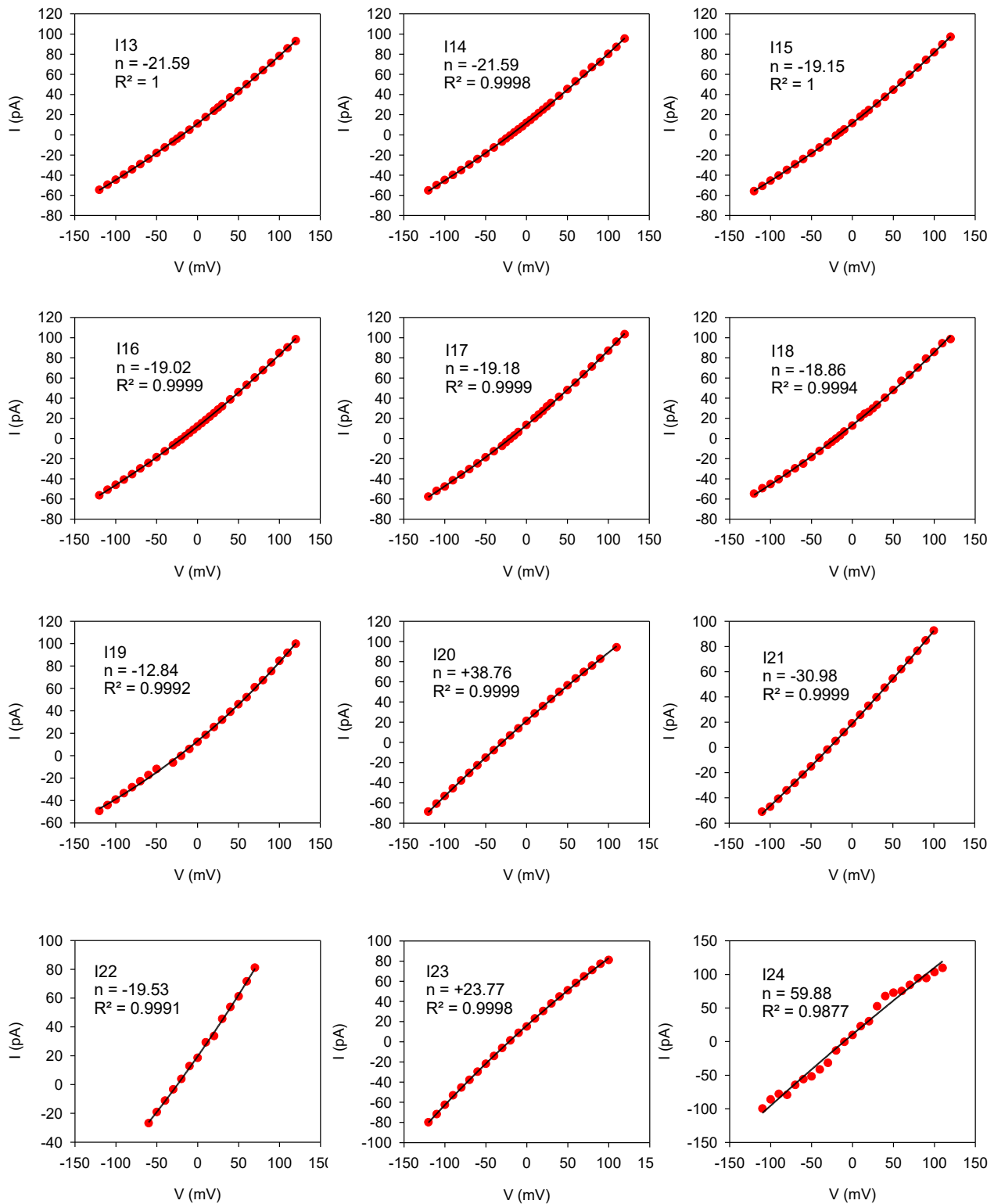

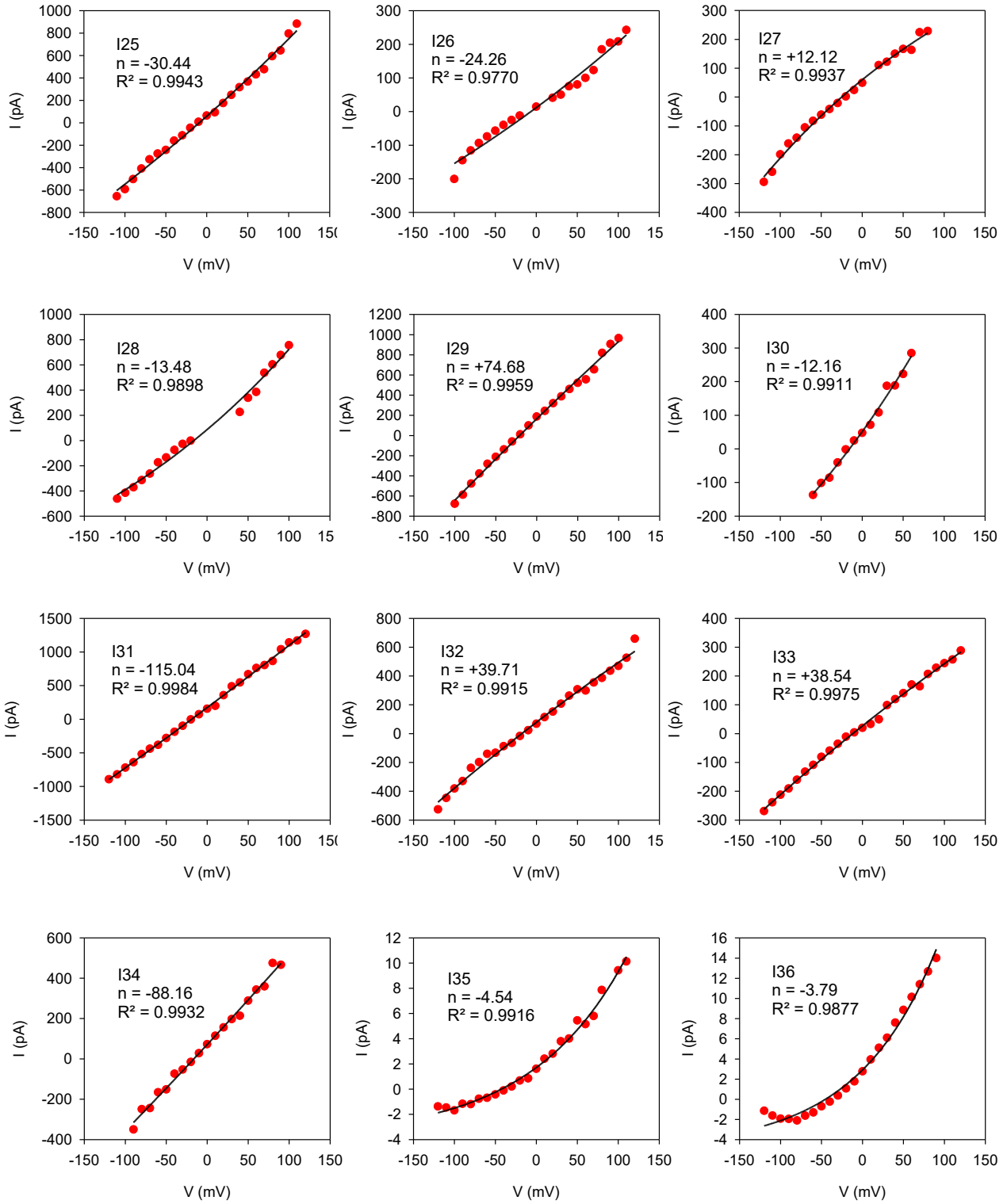

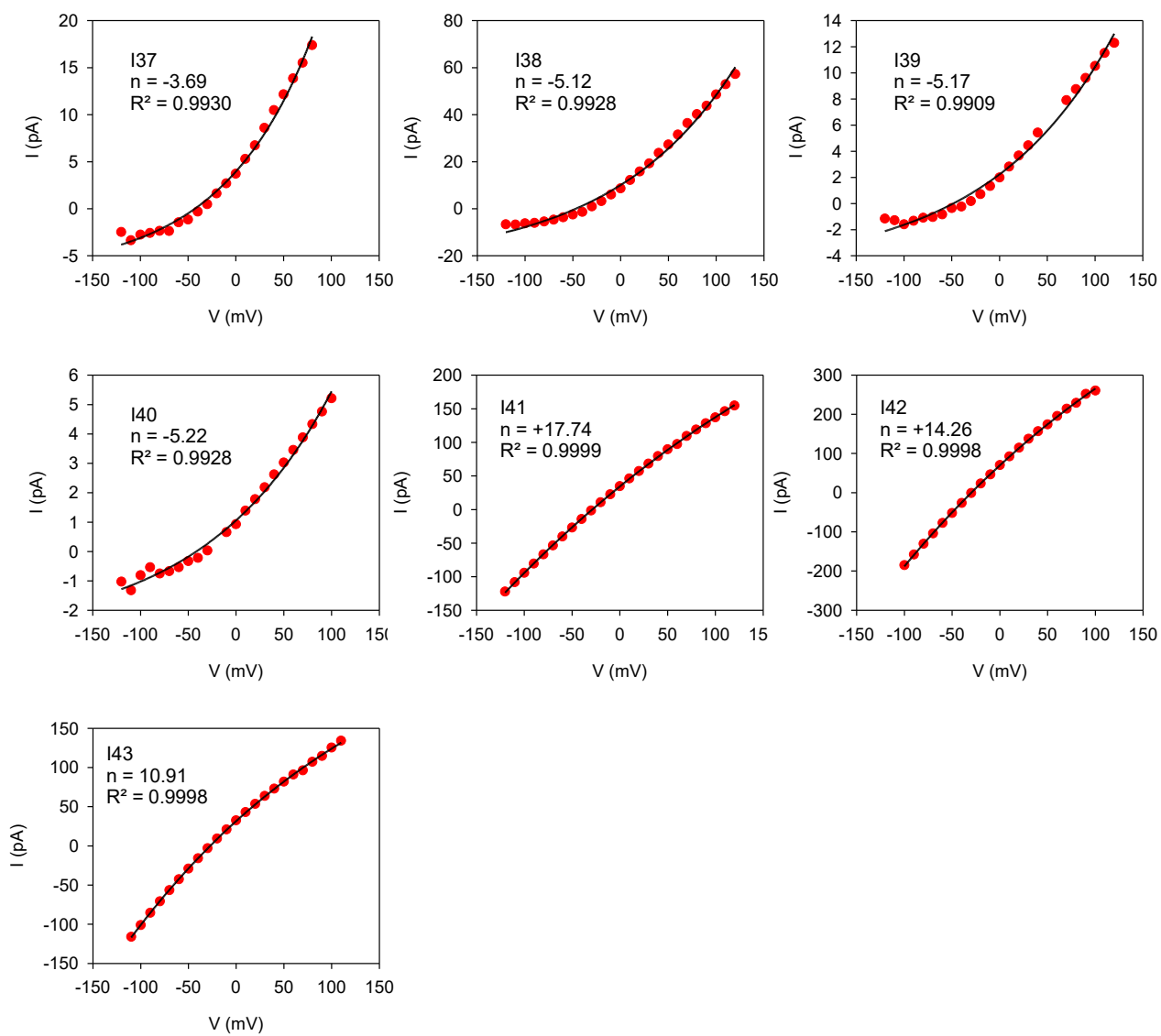

Supplement: S3 Fig — Each I-V curve (red circles) correspond to an independent Tse4 insertion (n = 43, indicated as I1-I43). Solid lines correspond to the fitting of equation I∝[exp(−eVnkbT)−1] (see Methods for details). The ideality factor n and R² are shown for each I-V curve. Insertions 35–40 yield |n| < 10, thus being classified as non-ohmic, while the rest are ohmic, with |n| > 10. Moreover, the rectifying currents all have a negative n, indicating that they correspond to outward rectifying channels (see Methods for details). (PDF) [file ppat.1012981.s004.pdf]

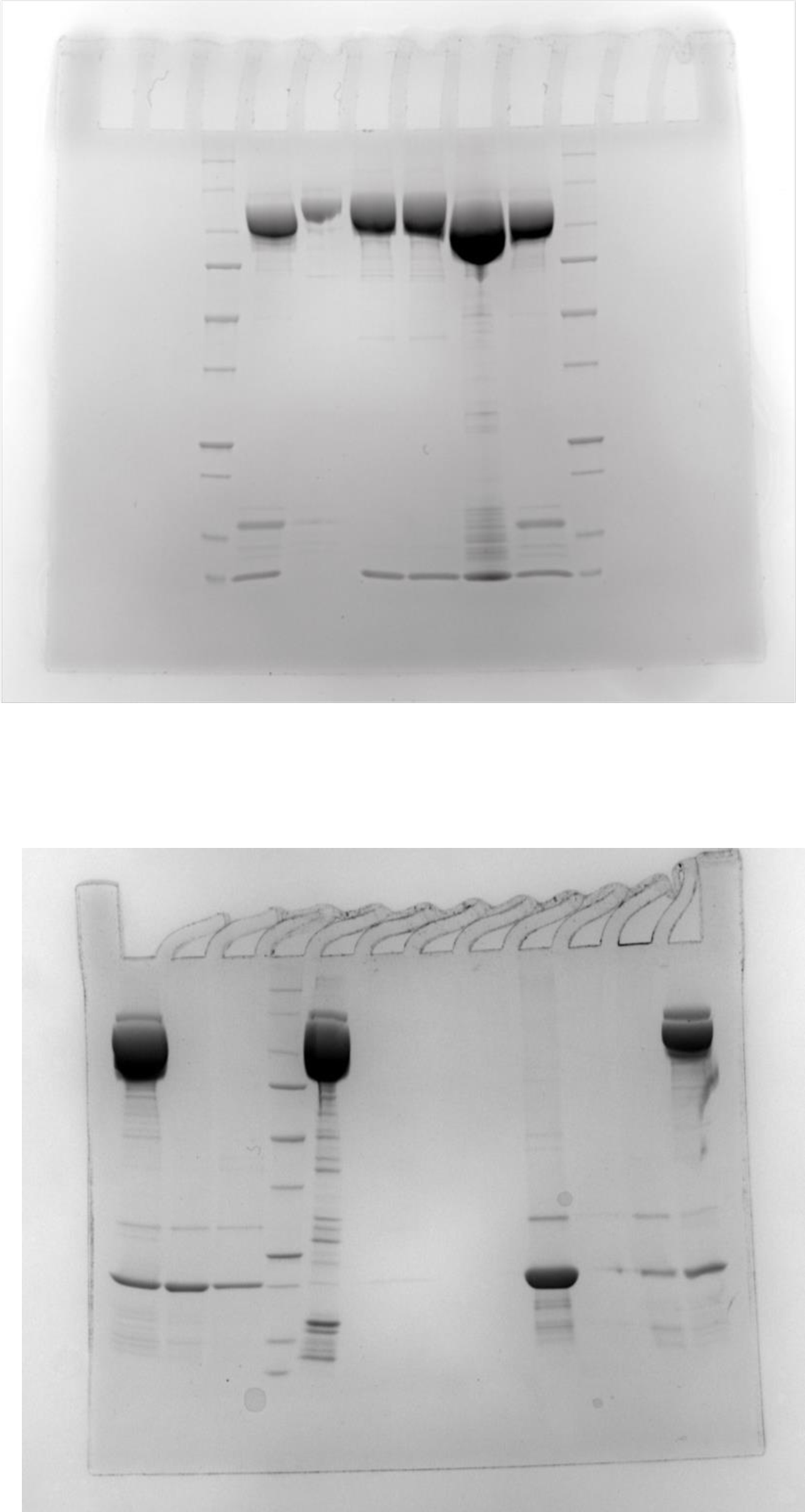

Supplement: S4 Fig — (TIF) [file ppat.1012981.s005.tif]
